# PET radiomics-based lymphovascular invasion prediction in lung cancer using multiple segmentation and multi-machine learning algorithms

**DOI:** 10.1007/s13246-024-01475-0

**Published:** 2024-09-03

**Authors:** Seyyed Ali Hosseini, Ghasem Hajianfar, Pardis Ghaffarian, Milad Seyfi, Elahe Hosseini, Atlas Haddadi Aval, Stijn Servaes, Mauro Hanaoka, Pedro Rosa-Neto, Sanjeev Chawla, Habib Zaidi, Mohammad Reza Ay

**Affiliations:** 1 Translational Neuroimaging Laboratory, The McGill University Research Centre for Studies in Aging, Douglas Hospital, McGill University, Montréal, Québec Canada; 2 Department of Neurology and Neurosurgery, Faculty of Medicine, McGill University, Montréal, Québec Canada; 3 Rajaie Cardiovascular Medical and Research Center, Iran University of Medical Science, Tehran, Iran; 4 Chronic Respiratory Diseases Research Center, National Research Institute of Tuberculosis and Lung Diseases (NRITLD), Shahid Beheshti University of Medical Sciences, Tehran, Iran; 5 PET/CT and cyclotron center, Masih Daneshvari Hospital, Shahid Beheshti University of Medical Sciences, Tehran, Iran; 6 Department of Medical Physics and Biomedical Engineering School of Medicine, Tehran University of Medical Sciences, Tehran, Iran; 7 Research Center for Molecular and Cellular Imaging (RCMCI), Advanced Medical Technologies and Equipment Institute (AMTEI), Tehran University of Medical Sciences (TUMS), Tehran, Iran; 8 Department of Electrical and Computer Engineering, Kharazmi University, Tehran, Iran; 9 School of Medicine, Mashhad University of Medical Science, Mashhad, Iran; 10 Department of Radiology, Perelman School of Medicine, University of Pennsylvania, Philadelphia, USA; 11 Division of Nuclear Medicine and Molecular Imaging, Geneva University Hospital, Geneva, 1211 Switzerland; 12 Department of Nuclear Medicine and Molecular Imaging, University of Groningen, University Medical Center, Groningen, 9700 RB Netherlands; 13 Department of Nuclear Medicine, University of Southern Denmark, Odense, 500 Denmark; 14 University Research and Innovation Center, Óbuda University, Budapest, Hungary

**Keywords:** PET, NSCLC, Lymphovascular invasion, Machine learning, Segmentation, Radiomics

## Abstract

The current study aimed to predict lymphovascular invasion (LVI) using multiple machine learning algorithms and multi-segmentation positron emission tomography (PET) radiomics in non-small cell lung cancer (NSCLC) patients, offering new avenues for personalized treatment strategies and improving patient outcomes. One hundred and twenty-six patients with NSCLC were enrolled in this study. Various automated and semi-automated PET image segmentation methods were applied, including Local Active Contour (LAC), Fuzzy-C-mean (FCM), K-means (KM), Watershed, Region Growing (RG), and Iterative thresholding (IT) with different percentages of the threshold. One hundred five radiomic features were extracted from each region of interest (ROI). Multiple feature selection methods, including Minimum Redundancy Maximum Relevance (MRMR), Recursive Feature Elimination (RFE), and Boruta, and multiple classifiers, including Multilayer Perceptron (MLP), Logistic Regression (LR), XGBoost (XGB), Naive Bayes (NB), and Random Forest (RF), were employed. Synthetic Minority Oversampling Technique (SMOTE) was also used to determine if it boosts the area under the ROC curve (AUC), accuracy (ACC), sensitivity (SEN), and specificity (SPE). Our results indicated that the combination of SMOTE, IT (with 45% threshold), RFE feature selection and LR classifier showed the best performance (AUC = 0.93, ACC = 0.84, SEN = 0.85, SPE = 0.84) followed by SMOTE, FCM segmentation, MRMR feature selection, and LR classifier (AUC = 0.92, ACC = 0.87, SEN = 1, SPE = 0.84). The highest ACC belonged to the IT segmentation (with 45 and 50% thresholds) alongside Boruta feature selection and the NB classifier without SMOTE (ACC = 0.9, AUC = 0.78 and 0.76, SEN = 0.7, and SPE = 0.94, respectively). Our results indicate that selection of appropriate segmentation method and machine learning algorithm may be helpful in successful prediction of LVI in patients with NSCLC with high accuracy using PET radiomics analysis.

**Supplementary Information:**

The online version contains supplementary material available at 10.1007/s13246-024-01475-0.

## Introduction

Lung cancer has the highest death rate of any cancer globally, and Non-Small Cell Lung Cancer (NSCLC) constitutes about 80–85% of all lung cancer cases [1]. Radical surgical resection is frequently the chosen treatment option for early-stage NSCLC [2]. However, systemic medicines like chemotherapy or immunotherapy are frequently advised in cases where NSCLCs are considered unresectable because of advanced stage or patient comorbidity, highlighting the customized approach required for the best possible lung cancer care [3]. The 5-year survival rate for early-stage NSCLC is approximately 30– 60% [4]. Lymphovascular invasion (LVI), defined by the presence of malignant tumor cells within endothelium-lined spaces of lymph or blood vessels, is the major prerequisite for tumor progression and distant metastasis development [5]. LVI is considered an independent negative prognostic indicator for loco-regional recurrence, poor disease-free, or overall survival outcomes in NSCLC patients [6]. Preoperative neoadjuvant chemotherapy and lobectomy with expanded lymph node dissection have been shown to be effective treatment strategies for NSCLC patients harboring LVI [7]. Therefore, early detection of LVI in NSCLC patients is essential for prognostication and the selection of appropriate treatment options. Histologic classification has remained the gold standard for identifying lymphovascular invasion (LVI), a critical factor that significantly influences prognosis and treatment strategies in lung cancer. Despite its invasiveness, this method is crucial for accurate staging and guiding therapeutic interventions, underscoring the need for precise and less invasive diagnostic alternatives [8].

Preoperative identification of LVI in NSCLC patients is challenging due to the absence of reliable biomarkers or diagnostic tools in the clinical setting [9]. Although [^18^F]-2-Fluoro-2-deoxy-D-glucose ([^18^F]-FDG)-positron emission tomography (PET) is commonly used for the diagnosis of NSCLCs, routine FDG-PET images cannot detect small vascular invasions owing to spatial resolution limitations. The application of advanced radiomics techniques can significantly enhance the detection capabilities. Radiomics, a quantitative analysis of phenotypic characteristics of lesions and intratumoral heterogeneity through in-depth mining of imaging data, can be used to predict outcomes, diagnose, and prognose abnormalities, including lung cancers [10–13]. PET radiomics and CT texture analyses have been used to predict LVI in lung adenocarcinomas [14] and NSCLCs [15]. In addition, machine learning classifiers of PET radiomics have been used to diagnose histological subtypes of lung cancers [16], brain tumors [17], esophageal cancers [18], and gastric cancer [19] with high accuracy.

However, various factors can affect the accuracy of predictions, [20–22], especially image segmentation methods [23]. A recent phantom study examined the impact of manual contouring variability on PET radiomic features and revealed the susceptibility of radiomic features to different segmentations [24]. In addition, Lu et al. studied the effects of segmentation and discretization on PET radiomic features, highlighting that only half of the features were robust against various segmentation methods [25]. Therefore, any slight modification in the quantitative analysis may alter the prediction accuracy. In contrast, other studies have demonstrated the potential of machine learning and radiomic features in predicting LVI and lymph node metastasis of different types of cancer, including lung cancer, gastric cancer, and cervical cancer. Li et al. [26] used a combination of a machine learning model of radiomic features, Cox-2, and Tenascin C expression to predict LVI in PET/CT radiomics with high accuracy. The highest performance of the area under the curve (AUC) reported in this study was greater than 0.91, suggesting the potential of machine learning in predicting LVI in PET radiomics. In a recent study, lymph node metastasis of gastric cancer was predicted using machine learning models, resulting in more than 0.95 accuracy [27]. In another study, Hua et al. [28] explored a technique for deep feature learning and multiparametric MRI-based radiomics for preoperative LVI prediction in early-stage cervical cancer. Both tumor and peritumor tissues had their radiomic features retrieved, and a deep learning model was developed using information from a training cohort of 111 patients. The AUCs achieved by the final model, which combined five radiomics and three deep learning features, were 0.77 for the validation cohort. The work highlights the opportunity for enhanced LVI prediction in early-stage cervical cancer using a combination of radiomics and deep learning methods.

Despite LVI being a recognized independent adverse prognostic factor, its detection is not typically incorporated into routine clinical practice. Furthermore, the current literature lacks sufficient focus on leveraging PET radiomic features and machine learning methodologies for predicting LVI. This conspicuous gap, which intertwines clinical practice and research, underscores the critical need for additional investigation in this domain to enhance prognostic accuracy and, ultimately, patient outcomes. In this study, a range of segmentation techniques were applied to FDG-PET images to identify the optimal segmentation method. Furthermore, diverse feature selection techniques and machine learning algorithms were employed to detect LVI in NSCLC patients, utilizing PET radiomic features extracted from regions of interest.

## Materials and methods

**Fig. 1:**
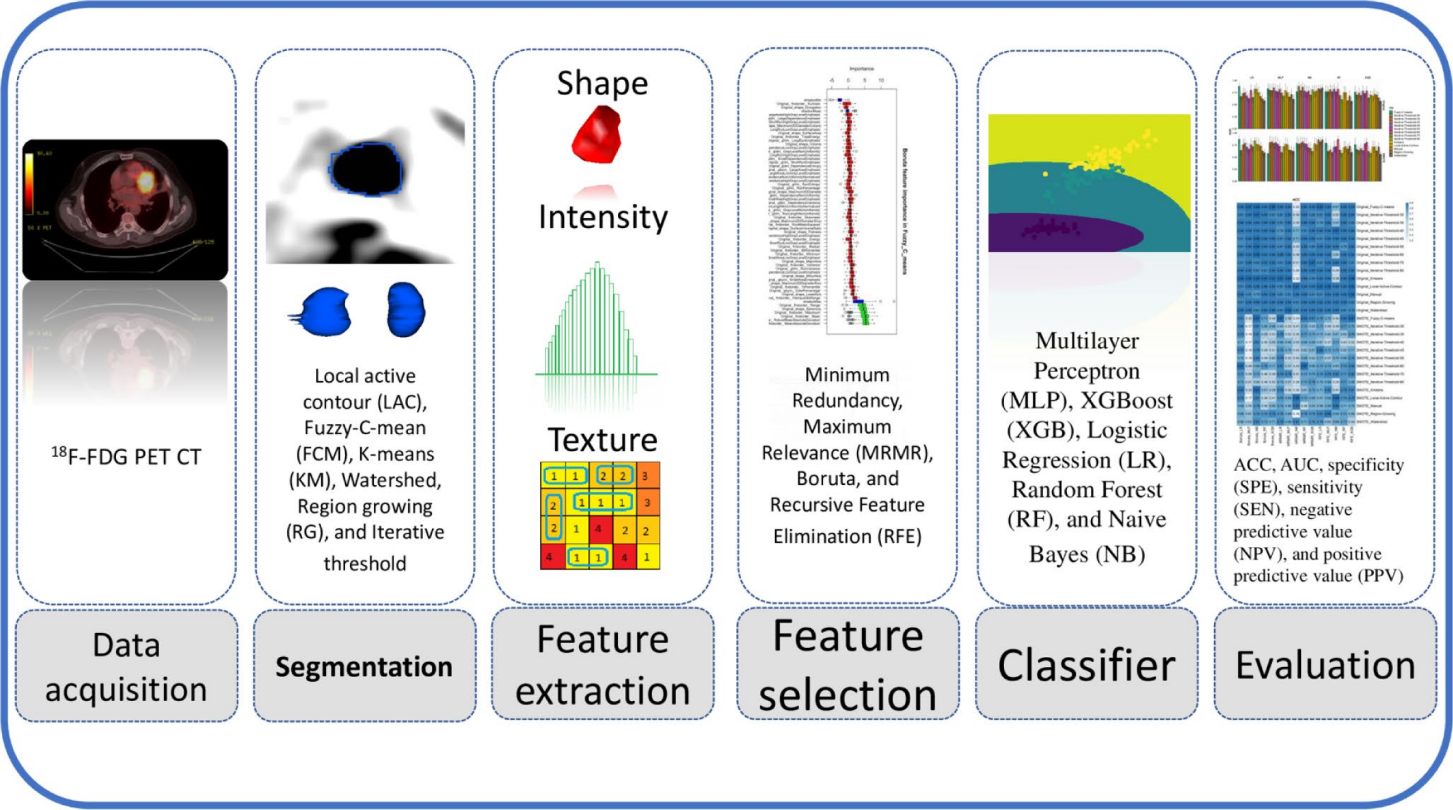
The framework adopted in the current study encompasses various steps from data acquisition to the evaluation of multiple machine learning classifiers

The methodology adopted in the current study involved a radiomics framework, as depicted in Fig. 1.

The first step was data acquisition, which involved obtaining FDG-PET images of NSCLC patients. Various image segmentation techniques were then applied to PET images to determine the best segmentation method. Next, regions of interest (ROIs) were defined based on the segmentations, and PET radiomic features extracted from the defined ROIs. Feature selection methods were applied to reduce the dimensionality of the data and select the most relevant features for predicting LVI. Multiple machine learning classifiers, such as logistic regression, support vector machines, and random forests, were then trained on the selected features and evaluated using cross-validation. The performance of the classifiers was assessed using metrics such as accuracy, sensitivity, specificity, and area under the receiver operating characteristic curve (AUC-ROC). Finally, the results were analyzed, and the most accurate classifier was selected for predicting LVI in NSCLC patients using PET radiomics features.

### Patient population and PET imaging

This retrospective study was approved by the Institutional Review Board (IRB) of Tehran University of Medical Sciences (approval ID IR.TUMS.MEDICINE.REC.1397.733). Due to the retrospective nature of the study, the requirement for obtaining written informed consent from patients was waived by the IRB. A cohort of 126 treatment-naive patients, consisting of 76 (60.4%) males and 50 (39.6%) females with a mean age of 47 ± 12 years were recruited. All patients had a biopsy-confirmed diagnosis of non-small cell lung cancer (NSCLC), with 36 (28.6%) patients showing LVI involvement and 90 (71.4%) patients showing no evidence of LVI involvement on histopathology. All patients in this cohort underwent a uniform treatment protocol. Each individual in the study, comprising both male and female patients, received the same initial treatment approach. This uniformity involved surgery as the first line of treatment, followed by a standardized post-operative care protocol, ensuring that the impact of different treatment modalities on LVI was minimized.

All patients included in this study underwent ^18^F-Fluorodeoxyglucose positron emission tomography/computed tomography (^18^F-FDG-PET/CT) imaging as part of their standard of care treatment, following a standard protocol. Prior to ^18^F-FDG-PET imaging, patients were required to fast for at least 6 h, and their plasma glucose concentrations were monitored to ensure they remained below 200 mg/dl. PET imaging was performed 50 to 70 min after the intravenous injection of ^18^F-FDG. PET/CT imaging was conducted on a 40-slice Biograph hybrid PET/CT scanner (Siemens Healthineers, Erlangen, Germany). Low-dose CT imaging was used for attenuation correction and anatomical localization. PET data were reconstructed utilizing the ordered subset-expectation maximization (OSEM) iterative algorithm, employing 3 iterations and 18 subsets. This process resulted in an image matrix of 256 × 256, with each pixel covering an area of 3.906 mm². A Gaussian post-reconstruction smoothing filter with a full width at half maximum (FWHM) of 4.5 mm was applied. All images were generated using the same reconstruction algorithm to minimize the impact of pre- and post-processing on the validity of imaging data. Furthermore, it’s essential to note that all patients had their FDG PET/CT scans performed within a closely monitored time frame before surgical procedures. The time interval between these scans and the surgery was consistent across the cohort, thereby reducing the variability that might affect the predictive accuracy of radiomics in assessing LVI. This consistency in both treatment and diagnostic timing provides a more controlled and reliable context for evaluating the impact of various factors on the presence and extent of LVI in non-small cell lung cancer patients.

### PET image segmentation methods

**Fig. 2:**
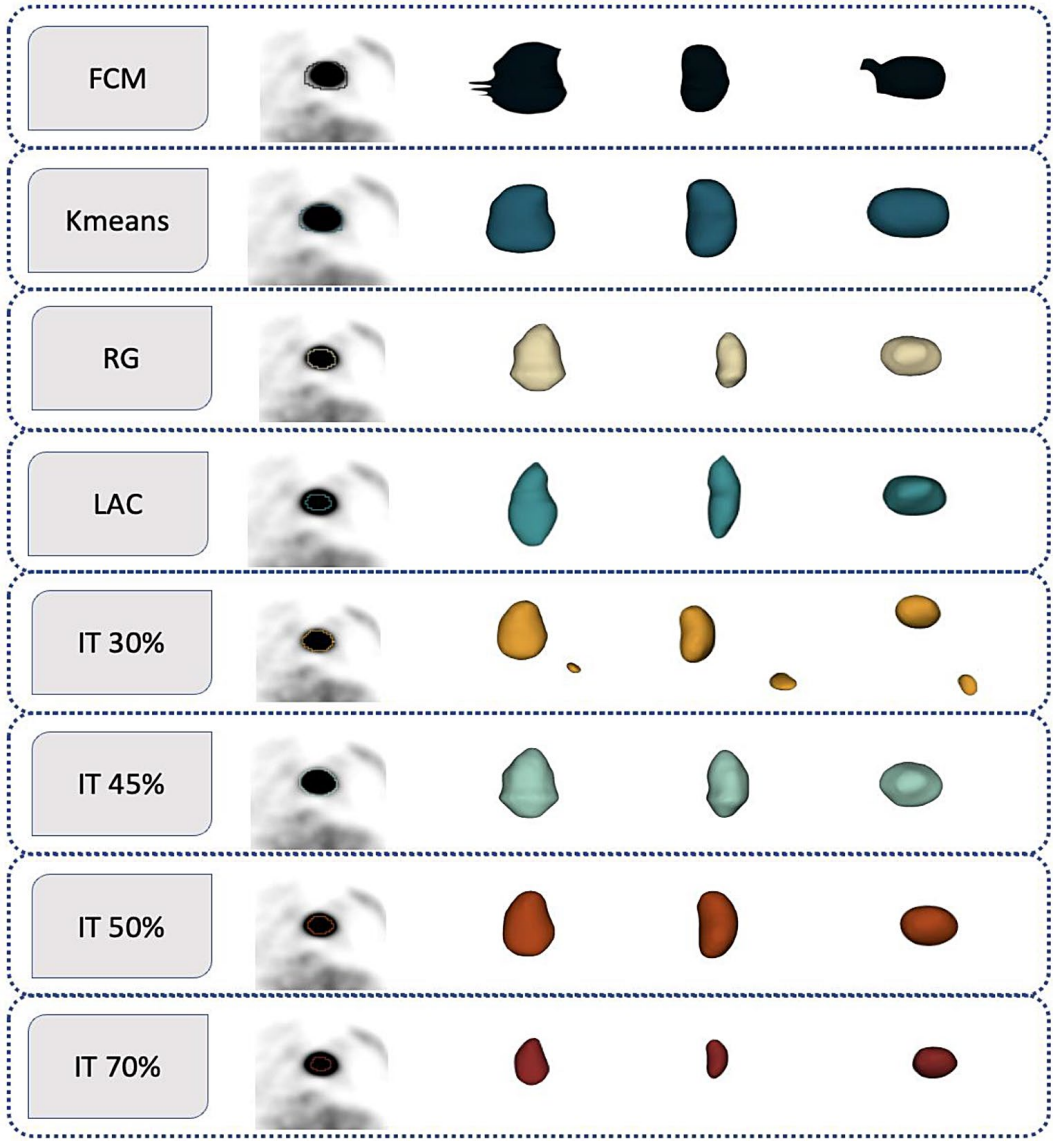
Multiple segmentation methods were applied to PET images, including Local active contour (LAC), Fuzzy-C-mean (FCM), K-means (KM), Region growing (RG), and Iterative thresholding (IT) with different percentages of the threshold. The central slice is shown in the first image on the left, the second one is the anterior view, the third one is the antero-posterior view, and the fourth superior-inferior view of one representative clinical study. The smooth appearance of the segmented volumes in this figure is a result of display smoothing applied for visualization purposes and does not reflect the actual voxel resolution

Various segmentation methods can be used in PET radiomic studies [29]. In our study, we implemented different PET image segmentation methods, including semi-automated and fully automated techniques that have been utilized more frequently, specifically for LVI in NCSCL. The segmentation methods used in this study were the Local Active Contour (LAC) [30], Fuzzy-C-mean (FCM) [31], K-means (KM) [32], Watershed [33], Region Growing (RG) [34], and Iterative Threshold (IT) [35], with different threshold percentage (30, 35, 40, 45, 50, 60, 70, and 80%). Using an in-house developed algorithm based on MATLAB 2022a software (Fig. 2), images were converted to ROIs for each of the 13 segmentation methods.

LAC Utilizes evolving contours within localized regions to capture the boundaries of lymphovascular invasion precisely [36]. FCM Implements soft clustering to assign each pixel a membership value for LVI, enabling smoother transitions between segmented areas [37]. KM utilizes hard clustering to partition image pixels into K clusters, often producing sharp-edged segmentation of LVI regions [38]. Watershed applies topological techniques to identify “catchment basins” and “watershed ridge lines” in the image, helping to segment intricate structures associated with LVI [39]. RG as semi-automated approach, starts from manually selected seed points and expands outward, aggregating pixels that meet specific criteria, effectively isolating areas indicative of LVI [40]. IT applies an iterative process to segment the image using different intensity levels, optimized for various threshold percentages (30%, 35%, 40%, 45%, 50%, 60%, 70%, 80%) to capture varying degrees of LVI visibility [41].

### Feature extraction

A total of 105 original radiomic features were extracted from each ROI delineated by the various segmentation methods explored in this study using the image biomarker standardization initiative (IBSI) [42] compliant Pyradiomics package [43]. These original radiomic features were derived from shape, first-order, second-order texture, and higher-order statistic features, including 13 shape features, 16 first-order statistical features, 23 Gy level co-occurrence matrix (GLCM) features, 14 Gy level dependence matrix (GLDM) features, 16 Gy level size zone matrix (GLSZM) features, 16 Gy level run length matrix (GLRLM) features, and 5 neighboring gray-tone difference matrix (NGTDM) features. From each patient, a total of 1365 imaging features (105 features using 13 different segmentation methods) were extracted. The details of these radiomic features are provided in supplementary Table 1.

### Feature selection

Given the high number of radiomic features, it is important to reduce the number of features to prevent overfitting. To achieve this, multiple feature selection algorithms were utilized, including minimum redundancy maximum relevance (mRmR), recursive feature elimination (RFE), and Boruta.

The dataset was carefully divided into mutually exclusive training (70%) and validation (30%) sets before any processing to prevent data leakage and ensure that the validation data remained unseen by the models during training. We employed a stratified split approach, maintaining the proportions of each class in the original dataset within both the training and validation sets. This method preserves the underlying distribution of the dataset and enhances the generalizability of our model. In our study, the mRmR feature selection algorithm was employed, resulting in the selection of a total of ten features. Unlike mRmR, RFE and Boruta feature selection algorithms were not constrained by a predetermined number of features. Instead, these methodologies dynamically determined the optimal quantity of features to be selected based on inherent algorithmic criteria, facilitating a more adaptive and potentially robust feature selection process.

### Classifier

Five machine learning classifiers were used to predict LVI, including Multilayer Perceptron (MLP), XGBoost (XGB), Logistic Regression (LR), Random Forest (RF), and Naive Bayes (NB).

MLP is a type of artificial neural network consisting of multiple layers of nodes in a directed graph [44]. It can model complex relationships between radiomic features and lymphovascular invasion status. XGBoost is an ensemble learning method aiming to optimize a sum of differentiable convex loss functions [45]. It can efficiently handle missing data and provides good predictive accuracy, making it useful in medical scenarios where some imaging data may be incomplete or noisy. LR is a statistical method for binary classification modeling the log-odds of the probability of the event [46]. It can provide a straightforward and interpretable model for predicting the likelihood of lymphovascular invasion based on radiomics features. RF is an ensemble learning method consisting of a multitude of decision trees, outputting the class reflecting the mode of the classes or mean prediction of the individual trees [47]. RF can handle a large number of features as input and provide an estimate of feature importance, which can be valuable for identifying key radiomic features related to lymphovascular invasion. NB classifiers are a family of probabilistic classifiers based on applying Bayes’ theorem with strong independence assumptions between the features [48]. NB is computationally efficient and could be used for initial rapid screening or in scenarios where computational resources are limited.

Due to the imbalanced nature of the dataset with regard to the two labels (LVI-positive and LVI-negative), the Synthetic Minority Oversampling Technique (SMOTE) was used to balance and improve prediction sensitivity. SMOTE is an oversampling technique that generates new artificial samples of the minority group by random oversampling, which helps preventing overfitting [49]. All the classifiers and the SMOTE algorithm were implemented using the mlr library [50] in R version 4.0.4 (The R Foundation, Vienna, Austria). To robustly evaluate the performance of each model, we assessed key metrics including accuracy (ACC), area under the curve (AUC), specificity (SPE), sensitivity (SEN), negative predictive value (NPV), and positive predictive value (PPV). These evaluations were conducted using a bootstrapping technique with 1000 iterations, allowing us to estimate the stability and reliability of these metrics under varying data conditions. Bootstrapping involved repeatedly sampling with replacement from the original validation set to generate multiple synthetic datasets. For each bootstrap sample, performance metrics were calculated, thereby accumulating a distribution of outcomes for each metric. This distribution was then used to compute 95% confidence intervals, offering insights into the variability and potential bias of the model’s performance estimates. The bootstrapping approach not only highlights the robustness of our models against different subsamples of data but also mitigates potential overfitting by demonstrating how the models might perform in genuinely unseen datasets. We did not hold out a subset of the dataset as a common test set for each algorithm after optimization because the primary focus was on maximizing the use of available data to ensure robust performance metrics through extensive resampling. The models were created based on one segmentation method, one feature selector, and one machine learning classifier, both with and without SMOTE, resulting in a total of 390 models (13 × 3 × 5 × 2).

## Results

**Fig. 3:**
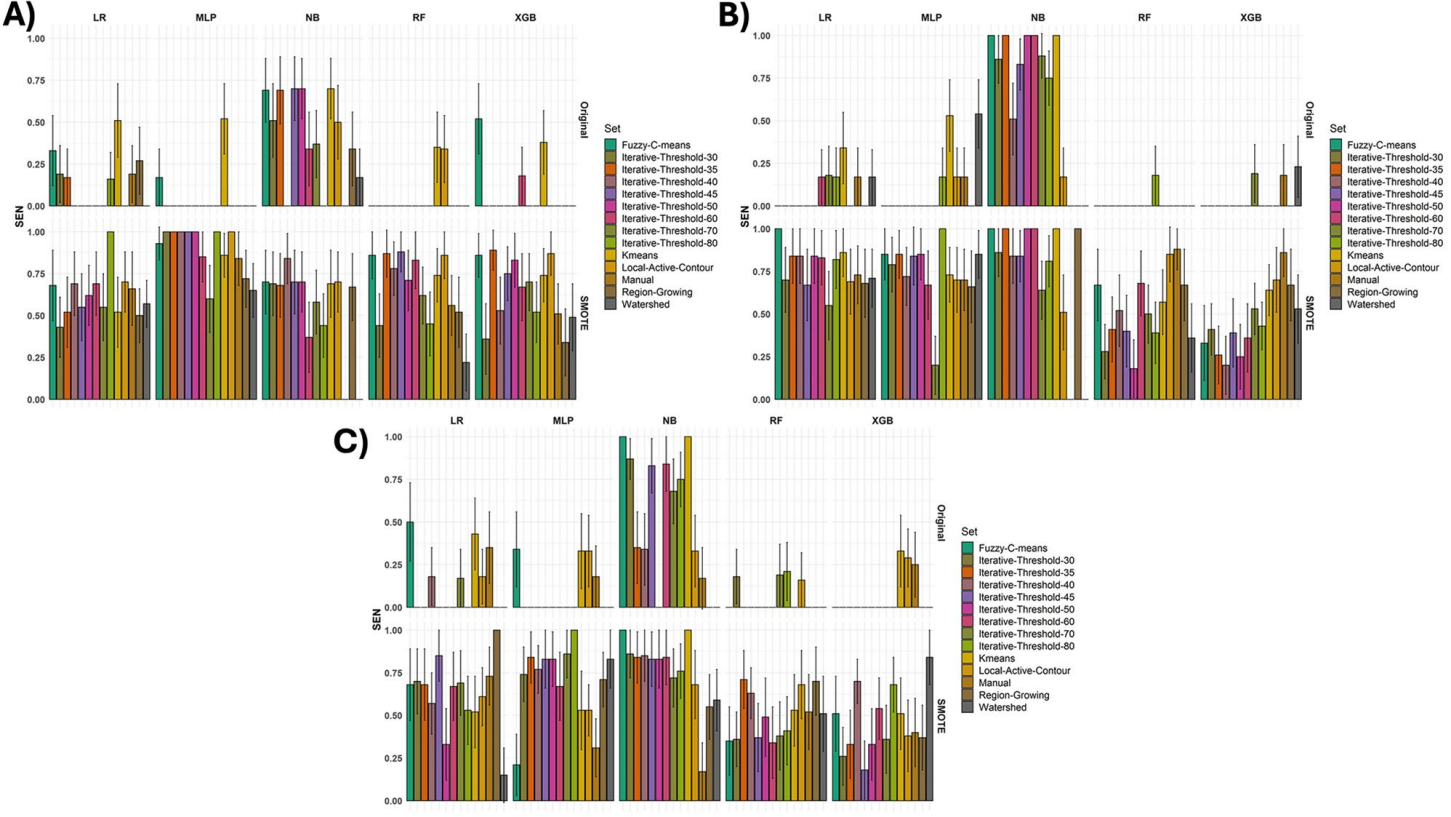
Sensitivity analysis of Boruta (**A**), MRMR (**B**), and RFE (**B**) feature selection over 13 image segmentation methods including LAC, FCM, K means, Watershed, RG, and iterative thresholding besides 5 machine learning classifiers including MLP, LR, XGB, NB, and RF. The results obtained with SMOTE (lower plot) and without SMOTE (upper plot) are shown

Figure 3 illustrates the sensitivity analysis of various feature selection methods across multiple segmentation techniques and machine learning algorithms used in this study. Bar plots A, B, and C correspond to Boruta, MRMR, and RFE feature selection methods, respectively. The lower plot shows the sensitivity values obtained after applying the SMOTE algorithm to balance the dataset, while the upper plot displays the original sensitivity values without using SMOTE. Our results demonstrate that the NB classifier, which uses distinct feature selection methods, exhibited a high level of sensitivity for LVI prediction. On the other hand, the MLP, RF, and XGB classifiers had the lowest sensitivity and were significantly improved after applying the SMOTE algorithm.

**Fig. 4:**
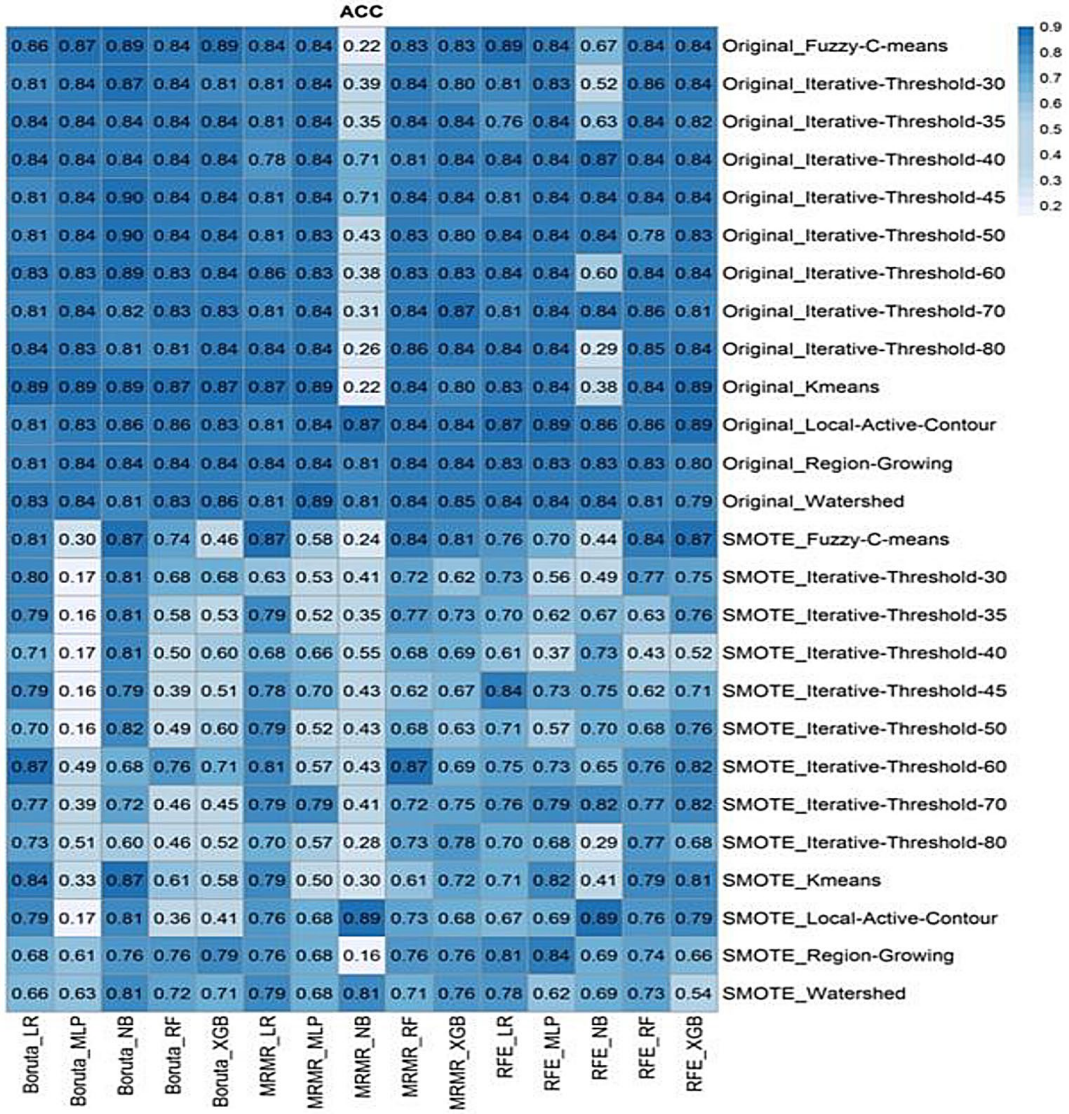
Accuracy (ACC) heatmap of multiple machine learning algorithms and feature selections over 13 image segmentations methods, including LAC, FCM, K means, Watershed, RG, and iterative thresholding besides 5 machine learning classifiers including MLP, LR, XGB, NB, and RF

Supplemental Figs. 1 and 2 present the ACC and AUC of different feature selectors applied to multiple machine learning algorithms and segmentation methods with and without SMOTE. The ACC of predicting LVI for different machine learning classifiers and feature selectors over various segmentation methods applied to PET images is summarized in Fig. 4. Additionally, supplemental Figs. 3–5 display the prediction power of AUC, SEN, and SPE, respectively, regarding different feature selection methods and classifiers over multiple segmentation methods examined in this study. The complete results of ACC, AUC, SEN, SPE, PPV, and NPV for each model are provided in Supplementary Table 2.

Overall, the ACC and AUC of LVI predictions reported in Supplemental Figs. 1 and 2 are promising, and the prediction power of all feature selections and classifiers is high. The SMOTE algorithm does not remarkably impact the AUC results. The FCM segmentation with RFE feature selection and NB classifier without SMOTE showed the highest predictive power of AUC (0.95). However, the LR and XBG classifiers had a lower predictive power among all classifiers examined in the study (Standard deviation (SD): 0.64 and 0.59, respectively). The prediction accuracy with all classifiers using Boruta feature selection was considerable, with more than 0.75 accuracy. The impact of the SMOTE algorithm on ACC results cannot be ignored, as it reduces the ACC, particularly in models with MLP classifier and Boruta feature selection (Supplemental Fig. 1A). The SMOTE NB classifier with Boruta feature selection had the lowest accuracy.

Table 1 sums up the results of the top ten AUC performances of the various machine learning classifiers and feature selection methods implemented in the current study. The AUC confidence interval (CI) of the 1000 bootstrapping method is reported in this table. Table 2 provides information regarding the top ten ACC performances of our models. Thirteen image segmentation methods, including LAC, FCM, K means, Watershed, RG, and iterative thresholding, were applied in this study. We aimed to choose the best model based on the classifiers and feature selection methods besides the segmentation algorithms.

**Table 1:** Top ten areas under the ROC curve (AUC) performances of our models for lymphovascular invasion prediction. The AUC confidence interval (CI) of 1000 bootstraps goes along with multiple feature selection (FS) methods, various segmentation (Seg) methods, and machine learning (ML) classifiers in the current study

Type	Seg	ML	FS	AUC	ACC	SEN	SPE	AUC CI
Original	FCM	NB	RFE	0.95	0.67	1	0.61	0.95–0.96
SMOTE	IT 45%	LR	RFE	0.93	0.84	0.85	0.84	0.92–0.93
SMOTE	FCM	LR	MRMR	0.92	0.87	1	0.84	0.92–0.92
Original	FCM	NB	MRMR	0.91	0.22	1	0.064	0.9–0.91
SMOTE	IT 50%	LR	MRMR	0.91	0.79	0.84	0.78	0.9–0.91
Original	Kmeans	NB	RFE	0.89	0.38	1	0.26	0.89–0.9
SMOTE	IT 45%	MLP	MRMR	0.89	0.7	0.84	0.67	0.88–0.89
SMOTE	IT 45%	MLP	RFE	0.89	0.73	0.83	0.71	0.89–0.9
SMOTE	IT 50%	NB	MRMR	0.88	0.43	1	0.32	0.88–0.89
SMOTE	IT 50%	NB	RFE	0.88	0.7	0.83	0.68	0.88–0.88

**Table 2:** Top ten accuracy (ACC) performances of our models for lymphovascular invasion prediction. The ACC confidence interval (CI) of 1000 bootstraps going along with multiple feature selection (FS) methods, various segmentation (Seg) methods, and machine learning (ML) classifiers in the current study

Type	Seg	ML	FS	ACC	AUC	SEN	SPE	ACC CI
Original	IT 45%	NB	Boruta	0.9	0.78	0.7	0.94	0.89–0.9
Original	IT 50%	NB	Boruta	0.9	0.76	0.7	0.94	0.88–0.90
SMOTE	LAC	NB	RFE	0.89	0.81	0.68	0.94	0.87–0.89
Original	FCM	NB	Boruta	0.89	0.77	0.69	0.93	0.87–0.89
Original	Kmeans	NB	Boruta	0.89	0.77	0.7	0.93	0.87–0.89
SMOTE	FCM	LR	MRMR	0.87	0.92	1	0.84	0.87–0.88
SMOTE	FCM	NB	Boruta	0.87	0.79	0.7	0.91	0.87–0.89
SMOTE	Kmeans	NB	Boruta	0.87	0.78	0.69	0.9	0.86–0.88
SMOTE	IT 60%	RF	MRMR	0.87	0.77	0.68	0.91	0.86–0.87
SMOTE	IT 60%	LR	Boruta	0.87	0.76	0.69	0.9	0.86–0.87

## Discussion

Early lung cancer detection remains challenging. Despite improvements in data acquisition methods [51], reconstruction algorithms [52], and analysis techniques, LVI is a known risk factor for poor prognosis and a recommendation for subsequent radiotherapy in many types of cancer [53]. Following NSCLC resection, LVI has been shown to independently predict early recurrence [7]. For certain gynecologic, head, and neck epithelial malignancies, adjuvant therapy may be considered even in node-negative illnesses when LVI is present [54].

Higashi et al. [55] used [^18^F]-FDG uptake as a predictor for LVI. They reported significant correlation with intertumoral lymphatic vessel invasion and lymph node metastasis. Our approach offers a more detailed analysis through image segmentation and feature extraction, leading to potentially higher predictive accuracy. Similarly, Li et al. [56] focused on volumetric metabolic parameters from preoperative [^18^F]-FDG PET/CT to predict primary tumor LVI, revealing metabolic tumor volume as an independent predictor. Our method integrates a broader range of radiomic features, aiming for a comprehensive evaluation. Wang et al.‘s study [57] on PET/CT radiomics for LVI prediction showed an AUC of 0.773, indicating the effectiveness of radiomics analysis. However, our approach aimed to refine prediction models further through advanced machine learning algorithms.

Hyun et al. [58] predicted the histological subtype of lung cancer with a machine learning approach, reporting that the LR model had the highest predictive power with an AUC of 0.85. In our study, the NB classifier with RFE feature selection and FCM segmentation showed the highest predictive power with an AUC of 0.95. A recent study involved a retrospective analysis of 112 patients who underwent PET/CT scans for early-stage cervical squamous cell cancer [59]. On the basis of PET/CT scans, 401 radiomic features were retrieved, and LVI was predicted using a combination of PET radiomics and a unique protein production. The strongest model for predicting LVI was a radiomics model with an AUC of 0.91, higher than the combined model (AUC = 0.80), demonstrating the remarkable ability of the machine learning model of PET radiomic features to predict LVI. Long et al. [60] investigated the potential of conventional MRI-based radiomics for predicting LVI in patients with endometrial cancer. The study’s results demonstrated a high level of predictive performance, yielding an AUC of 0.93 and an accuracy of 0.94. These remarkable metrics were derived from a comprehensive dataset comprised of 184 female patients. Zhou et al. [61] aimed to predict the lymph node metastasis of gastric cancer. They used seven machine learning algorithms on data from more than 1,000 patients and found that the Gradient Boosting Decision Trees classifier showed the highest accuracy, approximately 0.95. In our results, the NB classifier with Boruta feature selection and IT segmentation with a 45% threshold showed the highest accuracy (ACC = 0.90). Singh et al. [62], in a machine learning approach for detecting and classifying lung cancer, reported that the MLP classifier had the highest performance, achieving an ACC of 0.88. Hu et al. [63] predicted lymph node metastasis of NSCLC. Their study showed that the RF classifier had the highest AUC (0.83). The occurrence of imbalanced datasets represents a prevalent and significant hurdle in medical research, often posing complex challenges to the validity and generalizability of study outcomes. Collecting a fully balanced dataset may only sometimes be feasible in clinical practice. An oversampling algorithm, such as SMOTE, can be used to improve the prediction sensitivity.

In this study, we used the SMOTE algorithm to address the imbalance issue in our dataset. However, we found that the SMOTE algorithm had a negative impact on the accuracy and AUC results, especially in the case of the MLP classifier with Boruta feature selection. In Fig. 3, we illustrate the immediate effect of the SMOTE algorithm on sensitivity. We observed that the sensitivity of the original (without SMOTE) prediction was low, except for the NB classifier, which demonstrated higher sensitivity than other classifiers, particularly with MRMR feature selection. The RF classifier showed the lowest sensitivity, especially with the MRMR feature selection. In the lower plot of Fig. 3, the influence of SMOTE is readily apparent, significantly enhancing the sensitivity of LVI prediction. As a case in point, the MLP classifier, in conjunction with the Boruta feature selection method, showed a marked increment in sensitivity following the implementation of the SMOTE algorithm, underscoring the effectiveness of this resampling technique in imbalanced data scenarios.

In the current study, we compared the performance of different segmentation methods, machine learning algorithms, and feature selection methods of PET radiomic features in predicting LVI in NSCLC patients. We found a 10% variation between the highest AUC of FCM and IT-60% segmentation. Regarding accuracy, the Boruta feature selection demonstrated the highest performance (ACC = 0.90) with the IT-45% segmentation, while the RFE feature selection showed the highest predictive power (AUC = 0.95) of the FCM segmentation. Upon examination, selecting the model for segmentation, feature selection, and machine learning classifiers might have a 10 to 30% variation in the final results. Based on the feasibility of coding for the segmentation algorithms, iterative thresholding is one of the suitable methods with the highest accuracy in NSCLC PET images. In the radiomics-based machine learning study, the choice of feature selection algorithms and machine learning classifier might depend on the data set.

The SMOTE algorithm, by generating synthetic examples of the minority class, attempts to balance the distribution of the classes by providing more training samples for the minority class. This allows the machine learning algorithms to better capture the patterns and features of the minority class, resulting in higher sensitivity (the ability to identify positive cases correctly). Although SMOTE algorithm improves the sensitivity, it may negatively impact ACC and AUC in most machine learning classifiers. This is due to the fact that synthetic examples generated by SMOTE introduce some level of noise and may cause the model to become more prone to misclassifying the majority class. As a result, the overall accuracy and AUC may slightly decrease.

The trade-off between sensitivity, accuracy, and AUC should be carefully considered when applying the SMOTE algorithm. Depending on the specific requirements and priorities of the application, the decision to use SMOTE should be based on the relative importance of correctly identifying positive cases (sensitivity) versus overall accuracy and the balance between the two. In formulating the classification models of LVI in lung cancer using PET images, one major dilemma involves the weighing of different factors, including AUC, SEN, and SPE. For this kind of model, the AUC provides a good assessment of the extent to which the model is capable of accurately predicting the presence or absence of LVI across all the thresholds computed. However, there is a certain tension that exists between the sensitivity and specificity of a diagnostic model, as applied to concrete clinical situations important consequences follow from false negatives and false positives. In terms of LVI, a false negative result means that an LVI case is somehow missed, which could imply that the patient is not given the most appropriate therapeutic plan, making sensitivity a high-risk factor. On the other hand, high specificity is crucial for avoiding treatment interventions for disorders that are not present due to false positive results which in turn pose physical and psychological burden to the patients. A model with high sensitivity (true positive rate) is effective at correctly identifying patients. Therefore, it would flag fewer false negatives, meaning fewer patients who actually have LVI would be missed. Consequently, this could reduce the number of unnecessary biopsies, as patients flagged as negative by the model would have a high likelihood of not having LVI, thus reducing the need for further invasive procedures for those patients. Our discussion also addresses these trade-offs and specifies aspects in the present work where optimal equal balance between sensitivity and specificity was sought to avoid a costly exchange of one of these advantages for the other. It serves the clinician’s purpose of help in choosing a right model given the risk tolerances relevant to their clinical practice and the treatment thresholds a model needs to meet for the diagnostic information it is going to deliver to be clinically useful, as well as necessary.

The selected features are based on the combination of IT segmentation with a 45% threshold and RFE feature selection (supplemental Table 3) are mainly categorized in shape, intensity, and texture features family that are correlated with the likelihood of lymphovascular invasion in NSCLC patients, providing a basis for the prediction model. The selected features are organized into several categories: shape (Flatness, Maximum3D_Diameter, MajorAxis), first-order (Maximum, 90Percentile, Range, RobustMeanAbsoluteDeviation, MeanAbsoluteDeviation, RootMeanSquared, 10Percentile, Median, Mean, Minimum, Variance, InterquartileRange), and textural, which includes GLSZM SmallAreaLowGrayLevelEmphasis, GLSZM SmallAreaHighGrayLevelEmphasis, GLDM LargeDependenceEmphasis, and GLSZM SmallAreaEmphasis. Shape-based family (Flatness, Maximum3D_Diameter, MajorAxis) provide valuable information regarding its growth pattern, invasiveness, and potential response to treatment. According to the IBSI radiomic feature definition [42], tumors with irregular shapes or larger diameters might indicate more aggressive behavior. The ‘Flatness’ feature can provide insights into the overall 3D shape of the tumor, while ‘MajorAxis’ and ‘Maximum3D_Diameter’ measure the size and elongation of the tumor. The first-order family features are based on the distribution of voxel intensities within the tumor region and provide information about the overall intensity variations [42]. ‘Mean,’ ‘Median,’ ‘Variance,’ and other statistical measures describe the central tendency, spread, and distribution of voxel intensities. An uneven or skewed distribution of intensities might reflect the heterogeneity of the tumor, which could be linked to different biological characteristics, potentially impacting LVI. GLSZM (Gray Level Size Zone Matrix) features capture the relationship between the size of connected regions of similar intensity levels in the tumor [42]. ‘SmallAreaLowGrayLevelEmphasis’ and ‘SmallAreaHighGrayLevelEmphasis’ highlight the influence of small regions with low and high gray levels, respectively. These features could be linked to the texture and heterogeneity of the tumor, which in turn might be related to its aggressiveness and potential for invasion. GLDM (Gray Level Dependence Matrix) features characterize the dependence between pairs of voxels based on their gray-level values [42]. ‘LargeDependenceEmphasis’ could reflect the presence of larger homogeneous regions within the tumor, potentially indicating a more organized or structured growth pattern [42].

Among the limitations of the current study was the low sample size, which prevented the implementation of deep learning algorithms for predicting LVI in NSCLC subjects. We did not hold out a common test set to maximize data use for robust performance metrics via resampling. In addition, deep learning fully automated-based segmentation methods were not implemented in the current study. Further investigation of their potential is guaranteed. Another limitation of this study is that the PET/CT scans were standardized, conducted within a tightly controlled timeframe before surgical procedures, and utilized uniform scanner settings and reconstruction parameters. While this approach strengthens the internal consistency of our radiomic analyses, it potentially limits the generalizability of our findings across different imaging conditions. Radiomic features, particularly texture-based ones, are known to be influenced by variations in acquisition and reconstruction parameters, scanner types, and noise levels. To ensure the broader applicability of our results, future studies should aim to validate these predictive models across multiple centers with diverse imaging setups. In addition, the observed fluctuations in sensitivity, ranging from classifying all or no cases as positive in some cases, may reflect both the variable nature of LVI presentation and limitations within our models. Enhancing model robustness and diversifying datasets could help stabilize these predictions. 

## Conclusion

The prediction of LVI in NSCLC patients is of great importance in medical treatment, and radiomics studies have shown promising results in this regard. Our findings suggest that the combination of IT segmentation with a 45% threshold, RFE feature selection, LR classifier, and SMOTE algorithm provides the highest accuracy in predicting LVI (AUC = 0.93, ACC = 0.84, SEN = 0.85, SPE = 0.84). It is noteworthy that the SMOTE algorithm can improve the sensitivity of the prediction in an imbalanced dataset but may have a minor negative impact on ACC and AUC in most classifiers. The results of this study demonstrate the potential of using radiomic features in predicting LVI in NSCLC patients. Further research with larger datasets and data augmentation techniques is recommended for validating our findings.

## Electronic supplementary material

Below is the link to the electronic supplementary material.

Supplementary Material 1

## Data Availability

The data underlying this study’s findings, as well as the data processing algorithms, will be made available by the investigative team upon reasonable request.
