# Supplementary material for: PET radiomics-based lymphovascular invasion prediction in lung cancer using multiple segmentation and multi-machine learning algorithms
Source: Phys Eng Sci Med. 2024 Sep 3;47(4):1613–25. doi: 10.1007/s13246-024-01475-0 (PMC11666702; doi:10.1007/s13246-024-01475-0)
Supplement: Supplementary file 1 — Supplementary Material 1 [file 13246_2024_1475_MOESM1_ESM.pdf]

Supplementary material

Table 1. Radiomics features' name, set, and family extracted in this study.

Set	Type	Name
**Original**	shape	Maximum3DDiameter
**Original**	shape	Maximum2DDiameterSlice
**Original**	shape	Sphericity
**Original**	shape	MinorAxis
**Original**	shape	Elongation
**Original**	shape	SurfaceVolumeRatio
**Original**	shape	Volume
**Original**	shape	MajorAxis
**Original**	shape	SurfaceArea
**Original**	shape	Flatness
**Original**	shape	LeastAxis
**Original**	shape	Maximum2DDiameterColumn
**Original**	shape	Maximum2DDiameterRow
**Original**	gldm	DependenceEntropy
**Original**	gldm	DependenceNonUniformity
**Original**	gldm	GrayLevelNonUniformity
**Original**	gldm	SmallDependenceEmphasis
**Original**	gldm	SmallDependenceHighGrayLevelEmphasis
**Original**	gldm	DependenceNonUniformityNormalized
**Original**	gldm	LargeDependenceEmphasis
**Original**	gldm	LargeDependenceLowGrayLevelEmphasis
**Original**	gldm	DependenceVariance
**Original**	gldm	LargeDependenceHighGrayLevelEmphasis
**Original**	gldm	SmallDependenceLowGrayLevelEmphasis
**Original**	firstorder	InterquartileRange
**Original**	firstorder	Skewness
**Original**	firstorder	Median
**Original**	firstorder	Energy
**Original**	firstorder	RobustMeanAbsoluteDeviation
**Original**	firstorder	MeanAbsoluteDeviation
**Original**	firstorder	TotalEnergy
**Original**	firstorder	Maximum
**Original**	firstorder	RootMeanSquared
**Original**	firstorder	90Percentile
**Original**	firstorder	Minimum
**Original**	firstorder	Range
**Original**	firstorder	Variance
**Original**	firstorder	10Percentile
**Original**	firstorder	Kurtosis

**Original**	firstorder	Mean
**Original**	glrlm	ShortRunLowGrayLevelEmphasis
**Original**	glrlm	RunVariance
**Original**	glrlm	GrayLevelNonUniformity
**Original**	glrlm	LongRunEmphasis
**Original**	glrlm	ShortRunHighGrayLevelEmphasis
**Original**	glrlm	RunLengthNonUniformity
**Original**	glrlm	ShortRunEmphasis
**Original**	glrlm	LongRunHighGrayLevelEmphasis
**Original**	glrlm	RunPercentage
**Original**	glrlm	LongRunLowGrayLevelEmphasis
**Original**	glrlm	RunEntropy
**Original**	glrlm	RunLengthNonUniformityNormalized
**Original**	glszm	LargeAreaEmphasis
**Original**	glszm	SmallAreaHighGrayLevelEmphasis
**Original**	glszm	ZonePercentage
**Original**	glszm	LargeAreaLowGrayLevelEmphasis
**Original**	glszm	LargeAreaHighGrayLevelEmphasis
**Original**	glszm	SmallAreaEmphasis
**Original**	glszm	SmallAreaLowGrayLevelEmphasis

Table 2. Accuracy (ACC), area under the curve (AUC), sensitivity (SEN), specificity (SPE), positive predictive value (PPV), and negative predictive value (NPV) of predicting LI, belong to each feature selection methods and machine learning classifiers.

Type	AUC	ACC	SEN	SPE	PPV	NPV
Orginal_Fuzzy-C-means_RFE_NB	0.95	0.67	1	0.61	0.33	1
Orginal_Fuzzy-C-means_MRMR_MLP	0.94	0.84	0	1	0.36	0.84
SMOTE_Iterative-Threshold-45_RFE_LR	0.93	0.84	0.85	0.84	0.51	0.96
SMOTE_Fuzzy-C-means_MRMR_LR	0.92	0.87	1	0.84	0.55	1
SMOTE_Fuzzy-C-means_MRMR_GB	0.92	0.43	1	0.33	0.22	1
Orginal_Fuzzy-C-means_MRMR_NB	0.91	0.22	1	0.064	0.17	1
Orginal_Fuzzy-C-means_MRMR_GB	0.91	0.84	0	1	0.35	0.84
Orginal_Iterative-Threshold-45_RFE_LR	0.91	0.81	0	0.97	0.14	0.83
Orginal_Local-Active-Contour_RFE_KNN	0.91	0.87	0.16	1	0.86	0.86
SMOTE_Iterative-Threshold-50_MRMR_LR	0.91	0.79	0.84	0.78	0.42	0.96
Orginal_Local-Active-Contour_MRMR_NB	0.9	0.87	0.17	1	0.76	0.86
Orginal_Iterative-Threshold-45_MRMR_GB	0.89	0.84	0	1	0.96	0.84
Orginal_Kmeans_RFE_NB	0.89	0.38	1	0.26	0.2	1
SMOTE_Iterative-Threshold-45_MRMR_LR	0.89	0.78	0.67	0.8	0.4	0.93
SMOTE_Iterative-Threshold-45_MRMR_MLP	0.89	0.7	0.84	0.67	0.33	0.96
SMOTE_Iterative-Threshold-45_RFE_MLP	0.89	0.73	0.83	0.71	0.36	0.96
SMOTE_Iterative-Threshold-50_MRMR_GB	0.89	0.3	1	0.16	0.19	1
Orginal_Fuzzy-C-means_Boruta_LR	0.88	0.86	0.33	0.97	0.66	0.88
Orginal_Iterative-Threshold-30_Boruta_NB	0.88	0.87	0.51	0.94	0.61	0.91
Orginal_Manual_RFE_NB	0.88	0.86	0.17	1	0.76	0.86
SMOTE_Fuzzy-C-means_MRMR_LDA	0.88	0.84	0.67	0.87	0.5	0.93
SMOTE_Fuzzy-C-means_MRMR_RF	0.88	0.84	0.67	0.87	0.5	0.93
SMOTE_Iterative-Threshold-50_MRMR_NB	0.88	0.43	1	0.32	0.22	1
SMOTE_Iterative-Threshold-50_RFE_NB	0.88	0.7	0.83	0.68	0.33	0.95
SMOTE_Iterative-Threshold-80_MRMR_GB	0.88	0.27	1	0.13	0.18	1
SMOTE_Kmeans_RFE_NB	0.88	0.41	1	0.29	0.21	1
Orginal_Fuzzy-C-means_MRMR_LR	0.87	0.84	0	1	0.36	0.84
Orginal_Iterative-Threshold-50_MRMR_LR	0.87	0.81	0	0.97	0.34	0.83
Orginal_Iterative-Threshold-50_RFE_NB	0.87	0.84	0	1	0.68	0.84
Orginal_Iterative-Threshold-80_MRMR_MLP	0.87	0.84	0.17	0.97	0.48	0.86
SMOTE_Fuzzy-C-means_Boruta_LR	0.87	0.81	0.68	0.84	0.45	0.93
SMOTE_Iterative-Threshold-45_RFE_NB	0.87	0.75	0.83	0.74	0.38	0.96
SMOTE_Iterative-Threshold-50_RFE_SVM	0.87	0.89	0.67	0.94	0.66	0.94
SMOTE_Iterative-Threshold-60_Boruta_LDA	0.87	0.76	0.83	0.74	0.38	0.96
SMOTE_Iterative-Threshold-60_Boruta_RF	0.87	0.76	0.83	0.74	0.38	0.96
SMOTE_Region-Growing_RFE_LR	0.87	0.81	1	0.77	0.46	1
SMOTE_Region-Growing_RFE_GB	0.87	0.19	1	0.033	0.16	1
SMOTE_Watershed_RFE_MLP	0.87	0.62	0.83	0.58	0.27	0.95
Orginal_Fuzzy-C-means_RFE_LR	0.86	0.89	0.5	0.97	0.74	0.91
Orginal_Iterative-Threshold-40_Boruta_NB	0.86	0.84	0	1	0.91	0.84
Orginal_Iterative-Threshold-45_MRMR_LR	0.86	0.81	0	0.97	0.35	0.83

Orginal_Iterative-Threshold-45_RFE_MLP	0.86	0.84	0	1	0.29	0.84
Orginal_Iterative-Threshold-50_MRMR_MLP	0.86	0.83	0	1	0.72	0.83
Orginal_Watershed_RFE_LR	0.86	0.84	0	1	0.035	0.84
Orginal_Watershed_RFE_GB	0.86	0.84	0	1	0.035	0.84
Orginal_Watershed_RFE_MLP	0.86	0.84	0	1	0.035	0.84
SMOTE_Iterative-Threshold-35_MRMR_NB	0.86	0.35	1	0.23	0.2	1
SMOTE_Iterative-Threshold-60_MRMR_LR	0.86	0.81	0.83	0.81	0.46	0.96
SMOTE_Iterative-Threshold-60_RFE_LR	0.86	0.75	0.67	0.77	0.36	0.92
SMOTE_Iterative-Threshold-80_MRMR_MLP	0.86	0.57	1	0.49	0.27	1
SMOTE_Manual_Boruta_NB	0.86	0.78	0	0.94	0.11	0.83
SMOTE_Manual_Boruta_GB	0.86	0.16	1	0	0.16	NA
SMOTE_Manual_RFE_NB	0.86	0.86	0.17	1	0.99	0.86
SMOTE_Watershed_Boruta_NB	0.86	0.81	0	0.97	0.24	0.83
SMOTE_Watershed_RFE_GB	0.86	0.16	1	0	0.16	NA
Orginal_Iterative-Threshold-40_Boruta_MLP	0.85	0.84	0	1	0.89	0.84
Orginal_Iterative-Threshold-80_MRMR_GB	0.85	0.81	0	0.97	0.35	0.83
Orginal_Region-Growing_RFE_MLP	0.85	0.83	0	1	0.73	0.83
Orginal_Watershed_Boruta_NB	0.85	0.81	0.17	0.94	0.35	0.86
SMOTE_Fuzzy-C-means_RFE_NB	0.85	0.44	1	0.33	0.22	1
SMOTE_Iterative-Threshold-80_RFE_MLP	0.85	0.68	1	0.61	0.33	1
SMOTE_Region-Growing_MRMR_XGB	0.85	0.76	0.67	0.78	0.37	0.92
SMOTE_Watershed_RFE_LR	0.85	0.78	0.15	0.9	0.24	0.85
Orginal_Iterative-Threshold-45_RFE_GB	0.84	0.84	0	1	0.35	0.84
Orginal_Iterative-Threshold-50_MRMR_GB	0.84	0.81	0	0.97	0.34	0.83
Orginal_Iterative-Threshold-60_MRMR_NB	0.84	0.38	1	0.26	0.21	1
Orginal_Iterative-Threshold-80_RFE_GB	0.84	0.84	0	1	0.67	0.84
Orginal_Kmeans_RFE_MLP	0.84	0.84	0.33	0.93	0.49	0.88
SMOTE_Iterative-Threshold-30_MRMR_GB	0.84	0.3	1	0.16	0.19	1
SMOTE_Iterative-Threshold-45_MRMR_GB	0.84	0.3	1	0.16	0.19	1
SMOTE_Iterative-Threshold-60_MRMR_NB	0.84	0.43	1	0.32	0.22	1
SMOTE_Iterative-Threshold-80_Boruta_LR	0.84	0.73	1	0.68	0.38	1
SMOTE_Iterative-Threshold-80_MRMR_LR	0.84	0.7	0.82	0.68	0.32	0.95
Orginal_Fuzzy-C-means_RFE_GB	0.83	0.84	0.34	0.94	0.5	0.88
Orginal_Iterative-Threshold-45_RFE_NB	0.83	0.84	0.82	0.84	0.5	0.96
Orginal_Iterative-Threshold-60_RFE_LR	0.83	0.84	0	1	0.89	0.84
Orginal_Local-Active-Contour_MRMR_MLP	0.83	0.84	0.17	0.97	0.56	0.86
Orginal_Local-Active-Contour_RFE_NB	0.83	0.86	0.32	0.97	0.66	0.88
Orginal_Manual_Boruta_NB	0.83	0.78	0	0.93	0.081	0.83
Orginal_Manual_Boruta_GB	0.83	0.81	0	0.97	0.035	0.83
SMOTE_Fuzzy-C-means_RFE_LR	0.83	0.76	0.67	0.77	0.36	0.93
SMOTE_Iterative-Threshold-35_MRMR_LR	0.83	0.78	0.83	0.77	0.42	0.96
SMOTE_Iterative-Threshold-35_RFE_NB	0.83	0.67	0.83	0.64	0.31	0.95
SMOTE_Iterative-Threshold-45_RFE_GB	0.83	0.46	1	0.35	0.23	1
SMOTE_Iterative-Threshold-80_RFE_GB	0.83	0.35	1	0.23	0.2	1
SMOTE_Local-Active-Contour_RFE_KNN	0.83	0.73	0.67	0.75	0.34	0.92
SMOTE_Region-Growing_MRMR_GB	0.83	0.16	1	0	0.16	NA

Orginal_Fuzzy-C-means_Boruta_GB	0.82	0.92	0.49	1	0.95	0.91
Orginal_Fuzzy-C-means_Boruta_MLP	0.82	0.87	0.17	1	0.95	0.86
Orginal_Iterative-Threshold-80_Boruta_LR	0.82	0.84	0.16	0.97	0.46	0.86
Orginal_Region-Growing_Boruta_NB	0.82	0.84	0.34	0.94	0.51	0.88
Orginal_Region-Growing_MRMR_MLP	0.82	0.84	0	1	0.3	0.84
SMOTE_Fuzzy-C-means_MRMR_NB	0.82	0.24	1	0.097	0.18	1
SMOTE_Iterative-Threshold-30_RFE_GB	0.82	0.36	1	0.23	0.2	1
SMOTE_Iterative-Threshold-35_Boruta_MLP	0.82	0.16	1	0	0.16	NA
SMOTE_Iterative-Threshold-35_MRMR_GB	0.82	0.3	1	0.16	0.19	1
SMOTE_Iterative-Threshold-40_Boruta_NB	0.82	0.81	0.83	0.81	0.46	0.96
SMOTE_Iterative-Threshold-50_RFE_XGB	0.82	0.76	0.33	0.84	0.28	0.87
SMOTE_Iterative-Threshold-60_MRMR_GB	0.82	0.49	0.83	0.42	0.22	0.93
SMOTE_Local-Active-Contour_Boruta_KNN	0.82	0.81	0.83	0.81	0.46	0.96
SMOTE_Local-Active-Contour_MRMR_LDA	0.82	0.73	0.84	0.71	0.36	0.96
SMOTE_Local-Active-Contour_MRMR_RF	0.82	0.73	0.84	0.71	0.36	0.96
SMOTE_Local-Active-Contour_RFE_DT	0.82	0.65	0.84	0.62	0.3	0.95
SMOTE_Region-Growing_Boruta_NB	0.82	0.76	0.67	0.78	0.36	0.92
SMOTE_Region-Growing_MRMR_NB	0.82	0.16	1	0	0.16	NA
SMOTE_Region-Growing_MRMR_MLP	0.82	0.68	0.66	0.68	0.28	0.91
Orginal_Fuzzy-C-means_RFE_LDA	0.81	0.84	0	1	0.75	0.84
Orginal_Fuzzy-C-means_RFE_RF	0.81	0.84	0	1	0.75	0.84
Orginal_Iterative-Threshold-45_Boruta_LDA	0.81	0.84	0	1	0.35	0.84
Orginal_Iterative-Threshold-45_Boruta_RF	0.81	0.84	0	1	0.35	0.84
Orginal_Iterative-Threshold-45_MRMR_NB	0.81	0.7	0.82	0.68	0.33	0.95
Orginal_Iterative-Threshold-60_Boruta_MLP	0.81	0.84	0	1	0.74	0.84
Orginal_Iterative-Threshold-60_MRMR_LR	0.81	0.86	0.17	1	0.88	0.86
Orginal_Iterative-Threshold-80_Boruta_NB	0.81	0.81	0	0.97	0.25	0.83
Orginal_Local-Active-Contour_MRMR_GB	0.81	0.84	0	1	0.36	0.84
Orginal_Local-Active-Contour_RFE_SVM	0.81	0.84	0	1	0.32	0.84
Orginal_Region-Growing_RFE_NB	0.81	0.83	0	1	0.72	0.83
Orginal_Region-Growing_RFE_DT	0.81	0.81	0.16	0.94	0.33	0.85
Orginal_Watershed_RFE_DT	0.81	0.81	0.17	0.94	0.34	0.86
SMOTE_Fuzzy-C-means_MRMR_XGB	0.81	0.81	0.33	0.9	0.4	0.87
SMOTE_Iterative-Threshold-50_MRMR_MLP	0.81	0.52	0.84	0.45	0.23	0.93
SMOTE_Iterative-Threshold-50_RFE_MLP	0.81	0.57	0.83	0.52	0.25	0.94
SMOTE_Iterative-Threshold-60_Boruta_KNN	0.81	0.84	0.66	0.87	0.49	0.93
SMOTE_Kmeans_MRMR_GB	0.81	0.22	1	0.066	0.17	1
SMOTE_Region-Growing_MRMR_LR	0.81	0.76	0.67	0.78	0.36	0.92
SMOTE_Region-Growing_MRMR_LDA	0.81	0.76	0.67	0.78	0.37	0.93
SMOTE_Region-Growing_MRMR_RF	0.81	0.76	0.67	0.78	0.37	0.93
SMOTE_Watershed_MRMR_SVM	0.81	0.84	0.16	0.97	0.47	0.86
Orginal_Iterative-Threshold-30_Boruta_GB	0.8	0.81	0	0.97	0.13	0.83
Orginal_Iterative-Threshold-30_MRMR_MLP	0.8	0.84	0	1	0.11	0.84
Orginal_Iterative-Threshold-35_Boruta_LDA	0.8	0.84	0	1	0.52	0.84
Orginal_Iterative-Threshold-35_Boruta_RF	0.8	0.84	0	1	0.52	0.84
Orginal_Iterative-Threshold-50_MRMR_NB	0.8	0.43	1	0.32	0.23	1

Orginal_Iterative-Threshold-60_Boruta_LDA	0.8	0.84	0	1	0.91	0.84
Orginal_Iterative-Threshold-60_Boruta_RF	0.8	0.84	0	1	0.91	0.84
Orginal_Kmeans_Boruta_LR	0.8	0.89	0.5	0.97	0.73	0.91
Orginal_Kmeans_MRMR_NB	0.8	0.22	1	0.065	0.17	1
Orginal_Kmeans_RFE_GB	0.8	0.87	0.33	0.97	0.64	0.88
Orginal_Local-Active-Contour_RFE_LDA	0.8	0.87	0.16	1	0.89	0.86
Orginal_Local-Active-Contour_RFE_RF	0.8	0.87	0.16	1	0.89	0.86
Orginal_Manual_RFE_DT	0.8	0.86	0.34	0.97	0.67	0.88
Orginal_Watershed_RFE_LDA	0.8	0.81	0	0.97	0.048	0.84
Orginal_Watershed_RFE_RF	0.8	0.81	0	0.97	0.048	0.84
SMOTE_Fuzzy-C-means_RFE_XGB	0.8	0.87	0.51	0.94	0.59	0.91
SMOTE_Iterative-Threshold-40_Boruta_GB	0.8	0.16	1	0	0.16	NA
SMOTE_Iterative-Threshold-45_MRMR_DT	0.8	0.7	0.33	0.77	0.22	0.86
SMOTE_Iterative-Threshold-60_Boruta_XGB	0.8	0.71	0.66	0.71	0.31	0.92
SMOTE_Region-Growing_MRMR_SVM	0.8	0.81	0.34	0.91	0.41	0.88
Orginal_Fuzzy-C-means_RFE_MLP	0.79	0.84	0.34	0.94	0.49	0.88
Orginal_Iterative-Threshold-30_Boruta_SVM	0.79	0.84	0	1	0.34	0.84
Orginal_Iterative-Threshold-30_Boruta_MLP	0.79	0.84	0	1	0.68	0.84
Orginal_Iterative-Threshold-30_MRMR_GB	0.79	0.81	0	0.97	0.32	0.83
Orginal_Iterative-Threshold-30_RFE_MLP	0.79	0.83	0	1	0.35	0.83
Orginal_Iterative-Threshold-45_Boruta_KNN	0.79	0.79	0.17	0.91	0.27	0.85
Orginal_Iterative-Threshold-45_RFE_LDA	0.79	0.84	0	1	0.66	0.84
Orginal_Iterative-Threshold-45_RFE_RF	0.79	0.84	0	1	0.66	0.84
Orginal_Iterative-Threshold-60_Boruta_LR	0.79	0.84	0	1	0.76	0.84
Orginal_Iterative-Threshold-60_Boruta_KNN	0.79	0.86	0.17	1	0.76	0.86
Orginal_Iterative-Threshold-60_MRMR_LDA	0.79	0.83	0	1	0.29	0.83
Orginal_Iterative-Threshold-60_MRMR_RF	0.79	0.83	0	1	0.29	0.83
Orginal_Iterative-Threshold-60_MRMR_MLP	0.79	0.83	0	1	0.88	0.83
Orginal_Iterative-Threshold-80_Boruta_GB	0.79	0.81	0	0.97	0.044	0.83
Orginal_Iterative-Threshold-80_MRMR_KNN	0.79	0.78	0.33	0.87	0.33	0.87
Orginal_Region-Growing_RFE_LR	0.79	0.83	0	1	0.25	0.83
Orginal_Region-Growing_RFE_GB	0.79	0.83	0	1	0.25	0.83
Orginal_Watershed_MRMR_LDA	0.79	0.84	0	1	0.96	0.84
Orginal_Watershed_MRMR_RF	0.79	0.84	0	1	0.96	0.84
SMOTE_Iterative-Threshold-30_Boruta_KNN	0.79	0.81	0.5	0.87	0.43	0.9
SMOTE_Iterative-Threshold-60_RFE_MLP	0.79	0.73	0.67	0.74	0.33	0.92
SMOTE_Local-Active-Contour_RFE_NB	0.79	0.89	0.67	0.94	0.68	0.93
Orginal_Iterative-Threshold-30_RFE_GB	0.78	0.81	0	0.97	0.13	0.83
Orginal_Iterative-Threshold-35_Boruta_GB	0.78	0.84	0	1	0.88	0.84
Orginal_Iterative-Threshold-35_Boruta_SVM	0.78	0.84	0	1	0.37	0.84
Orginal_Iterative-Threshold-45_RFE_KNN	0.78	0.84	0	1	0.061	0.84
Orginal_Iterative-Threshold-60_Boruta_NB	0.78	0.89	0.34	1	0.88	0.89
Orginal_Iterative-Threshold-60_Boruta_XGB	0.78	0.84	0.17	0.97	0.51	0.86
Orginal_Iterative-Threshold-80_MRMR_DT	0.78	0.81	0.16	0.94	0.33	0.85
Orginal_Local-Active-Contour_Boruta_NB	0.78	0.86	0.49	0.94	0.6	0.9
Orginal_Local-Active-Contour_MRMR_XGB	0.78	0.84	0	1	0.87	0.84

Orginal_Region-Growing_MRMR_GB	0.78	0.84	0	1	0.91	0.84
SMOTE_Fuzzy-C-means_MRMR_MLP	0.78	0.57	0.84	0.52	0.25	0.94
SMOTE_Iterative-Threshold-30_Boruta_MLP	0.78	0.16	1	0	0.16	NA
SMOTE_Iterative-Threshold-30_RFE_NB	0.78	0.49	0.84	0.42	0.22	0.93
SMOTE_Iterative-Threshold-35_Boruta_GB	0.78	0.16	1	0	0.16	NA
SMOTE_Iterative-Threshold-40_Boruta_LR	0.78	0.71	0.68	0.71	0.32	0.92
SMOTE_Iterative-Threshold-40_Boruta_MLP	0.78	0.16	1	0	0.16	NA
SMOTE_Iterative-Threshold-40_MRMR_LR	0.78	0.68	0.83	0.65	0.31	0.95
SMOTE_Iterative-Threshold-40_RFE_NB	0.78	0.73	0.83	0.71	0.35	0.96
SMOTE_Iterative-Threshold-45_Boruta_MLP	0.78	0.16	1	0	0.16	NA
SMOTE_Iterative-Threshold-50_RFE_LR	0.78	0.71	0.33	0.78	0.22	0.86
SMOTE_Iterative-Threshold-50_RFE_GB	0.78	0.43	1	0.33	0.22	1
SMOTE_Iterative-Threshold-60_Boruta_SVM	0.78	0.78	0.66	0.81	0.4	0.93
SMOTE_Iterative-Threshold-60_MRMR_MLP	0.78	0.57	0.67	0.55	0.22	0.89
SMOTE_Iterative-Threshold-80_MRMR_KNN	0.78	0.87	0.67	0.9	0.56	0.94
SMOTE_Kmeans_MRMR_LR	0.78	0.78	0.83	0.78	0.41	0.96
SMOTE_Watershed_MRMR_NB	0.78	0.81	0	0.97	0.36	0.84
Orginal_Iterative-Threshold-35_Boruta_NB	0.77	0.84	0.67	0.87	0.5	0.93
Orginal_Iterative-Threshold-35_Boruta_MLP	0.77	0.84	0	1	0.88	0.84
Orginal_Iterative-Threshold-40_RFE_NB	0.77	0.87	0.34	0.97	0.68	0.88
Orginal_Iterative-Threshold-45_Boruta_XGB	0.77	0.84	0	1	0.24	0.84
Orginal_Iterative-Threshold-45_MRMR_MLP	0.77	0.84	0	1	0.31	0.84
Orginal_Iterative-Threshold-50_Boruta_LDA	0.77	0.84	0	1	0.53	0.84
Orginal_Iterative-Threshold-50_Boruta_KNN	0.77	0.81	0.16	0.94	0.37	0.85
Orginal_Iterative-Threshold-50_Boruta_RF	0.77	0.84	0	1	0.53	0.84
Orginal_Iterative-Threshold-60_Boruta_GB	0.77	0.84	0	1	0.34	0.84
Orginal_Iterative-Threshold-60_MRMR_GB	0.77	0.83	0	1	0.33	0.83
Orginal_Iterative-Threshold-60_RFE_NB	0.77	0.6	0.84	0.55	0.27	0.95
Orginal_Iterative-Threshold-70_RFE_NB	0.77	0.84	0.66	0.87	0.49	0.93
Orginal_Iterative-Threshold-80_RFE_SVM	0.77	0.81	0	0.97	0.35	0.84
Orginal_Kmeans_Boruta_MLP	0.77	0.89	0.5	0.97	0.74	0.91
Orginal_Kmeans_MRMR_LR	0.77	0.87	0.33	0.97	0.67	0.88
Orginal_Kmeans_RFE_LDA	0.77	0.84	0	1	0.87	0.84
Orginal_Kmeans_RFE_XGB	0.77	0.89	0.33	1	0.87	0.89
Orginal_Kmeans_RFE_RF	0.77	0.84	0	1	0.87	0.84
Orginal_Local-Active-Contour_RFE_MLP	0.77	0.89	0.32	1	0.9	0.89
Orginal_Region-Growing_MRMR_LR	0.77	0.84	0	1	0.96	0.84
SMOTE_Fuzzy-C-means_Boruta_NB	0.77	0.87	0.68	0.9	0.58	0.94
SMOTE_Fuzzy-C-means_Boruta_LDA	0.77	0.73	0.85	0.71	0.36	0.96
SMOTE_Fuzzy-C-means_Boruta_RF	0.77	0.73	0.85	0.71	0.36	0.96
SMOTE_Fuzzy-C-means_RFE_KNN	0.77	0.76	0.67	0.77	0.36	0.93
SMOTE_Iterative-Threshold-35_Boruta_NB	0.77	0.81	0.67	0.84	0.44	0.93
SMOTE_Iterative-Threshold-35_RFE_KNN	0.77	0.67	0.66	0.67	0.28	0.91
SMOTE_Iterative-Threshold-35_RFE_GB	0.77	0.32	1	0.19	0.19	1
SMOTE_Iterative-Threshold-45_Boruta_GB	0.77	0.16	1	0	0.16	NA
SMOTE_Iterative-Threshold-45_MRMR_NB	0.77	0.43	0.83	0.35	0.2	0.91

SMOTE_Iterative-Threshold-50_Boruta_XGB	0.77	0.6	0.83	0.55	0.26	0.94
SMOTE_Iterative-Threshold-80_RFE_KNN	0.77	0.78	0.49	0.84	0.37	0.89
SMOTE_Kmeans_RFE_KNN	0.77	0.81	0.66	0.84	0.44	0.93
SMOTE_Kmeans_RFE_SVM	0.77	0.84	0	1	0.24	0.84
SMOTE_Region-Growing_MRMR_DT	0.77	0.79	0.34	0.87	0.35	0.87
SMOTE_Watershed_MRMR_KNN	0.77	0.76	0.66	0.78	0.36	0.92
SMOTE_Watershed_MRMR_GB	0.77	0.16	1	0	0.16	NA
Orginal_Fuzzy-C-means_Boruta_KNN	0.76	0.87	0.5	0.93	0.59	0.91
Orginal_Iterative-Threshold-30_Boruta_LDA	0.76	0.84	0	1	0.67	0.84
Orginal_Iterative-Threshold-30_Boruta_KNN	0.76	0.81	0.34	0.91	0.42	0.88
Orginal_Iterative-Threshold-30_Boruta_RF	0.76	0.84	0	1	0.67	0.84
Orginal_Iterative-Threshold-45_Boruta_NB	0.76	0.9	0.68	0.94	0.67	0.94
Orginal_Iterative-Threshold-45_Boruta_GB	0.76	0.84	0	1	0.35	0.84
Orginal_Iterative-Threshold-45_Boruta_MLP	0.76	0.84	0	1	0.35	0.84
Orginal_Iterative-Threshold-45_MRMR_LDA	0.76	0.84	0	1	0.72	0.84
Orginal_Iterative-Threshold-45_MRMR_RF	0.76	0.84	0	1	0.72	0.84
Orginal_Iterative-Threshold-50_RFE_MLP	0.76	0.84	0	1	0.034	0.84
Orginal_Iterative-Threshold-60_RFE_GB	0.76	0.84	0	1	0.74	0.84
Orginal_Iterative-Threshold-60_RFE_MLP	0.76	0.84	0	1	0.73	0.84
Orginal_Iterative-Threshold-70_RFE_MLP	0.76	0.84	0	1	0.32	0.84
Orginal_Region-Growing_MRMR_NB	0.76	0.81	0	0.97	0.25	0.83
Orginal_Watershed_Boruta_GB	0.76	0.84	0	1	0.24	0.84
Orginal_Watershed_MRMR_NB	0.76	0.81	0	0.97	0.34	0.83
SMOTE_Fuzzy-C-means_Boruta_KNN	0.76	0.65	0.68	0.65	0.27	0.91
SMOTE_Fuzzy-C-means_Boruta_DT	0.76	0.44	0.85	0.36	0.2	0.92
SMOTE_Iterative-Threshold-30_RFE_LR	0.76	0.73	0.68	0.74	0.34	0.92
SMOTE_Iterative-Threshold-40_MRMR_GB	0.76	0.41	0.83	0.32	0.19	0.91
SMOTE_Iterative-Threshold-45_Boruta_NB	0.76	0.79	0.68	0.81	0.41	0.93
SMOTE_Iterative-Threshold-50_RFE_LDA	0.76	0.68	0.49	0.71	0.24	0.88
SMOTE_Iterative-Threshold-50_RFE_RF	0.76	0.68	0.49	0.71	0.24	0.88
SMOTE_Iterative-Threshold-60_Boruta_GB	0.76	0.19	1	0.033	0.17	1
SMOTE_Iterative-Threshold-60_MRMR_LDA	0.76	0.87	0.67	0.91	0.58	0.93
SMOTE_Iterative-Threshold-60_MRMR_RF	0.76	0.87	0.67	0.91	0.58	0.93
SMOTE_Iterative-Threshold-60_RFE_GB	0.76	0.43	1	0.32	0.22	1
SMOTE_Iterative-Threshold-80_Boruta_GB	0.76	0.19	1	0.032	0.17	1
SMOTE_Local-Active-Contour_MRMR_NB	0.76	0.89	0.49	0.97	0.73	0.91
SMOTE_Local-Active-Contour_RFE_LDA	0.76	0.76	0.67	0.78	0.37	0.92
SMOTE_Local-Active-Contour_RFE_RF	0.76	0.76	0.67	0.78	0.37	0.92
SMOTE_Watershed_RFE_DT	0.76	0.59	0.83	0.55	0.26	0.94
Orginal_Fuzzy-C-means_Boruta_NB	0.75	0.89	0.66	0.93	0.66	0.94
Orginal_Iterative-Threshold-40_Boruta_LR	0.75	0.84	0	1	0.65	0.84
Orginal_Iterative-Threshold-40_Boruta_LDA	0.75	0.84	0	1	0.31	0.84
Orginal_Iterative-Threshold-40_Boruta_GB	0.75	0.84	0	1	0.89	0.84
Orginal_Iterative-Threshold-40_Boruta_RF	0.75	0.84	0	1	0.31	0.84
Orginal_Iterative-Threshold-40_MRMR_LR	0.75	0.79	0	0.94	0.033	0.83
Orginal_Iterative-Threshold-50_Boruta_MLP	0.75	0.84	0	1	0.38	0.84

Orginal_Iterative-Threshold-50_RFE_LR	0.75	0.84	0	1	0.034	0.84
Orginal_Iterative-Threshold-50_RFE_GB	0.75	0.84	0	1	0.034	0.84
Orginal_Iterative-Threshold-70_MRMR_LDA	0.75	0.84	0	1	0.96	0.84
Orginal_Iterative-Threshold-70_MRMR_RF	0.75	0.84	0	1	0.96	0.84
Orginal_Iterative-Threshold-80_MRMR_LR	0.75	0.84	0.17	0.97	0.46	0.86
Orginal_Kmeans_Boruta_NB	0.75	0.89	0.67	0.93	0.66	0.94
Orginal_Kmeans_Boruta_GB	0.75	0.89	0.33	1	0.87	0.89
Orginal_Manual_RFE_GB	0.75	0.84	0.17	0.97	0.53	0.86
SMOTE_Fuzzy-C-means_Boruta_GB	0.75	0.21	1	0.064	0.17	1
SMOTE_Iterative-Threshold-30_Boruta_NB	0.75	0.81	0.67	0.84	0.45	0.93
SMOTE_Iterative-Threshold-30_Boruta_SVM	0.75	0.75	0.17	0.87	0.2	0.84
SMOTE_Iterative-Threshold-30_MRMR_NB	0.75	0.41	0.83	0.33	0.19	0.91
SMOTE_Iterative-Threshold-35_RFE_MLP	0.75	0.62	0.83	0.58	0.28	0.95
SMOTE_Iterative-Threshold-45_Boruta_KNN	0.75	0.7	0.34	0.77	0.23	0.86
SMOTE_Iterative-Threshold-50_Boruta_GB	0.75	0.16	1	0	0.16	NA
SMOTE_Iterative-Threshold-70_RFE_SVM	0.75	0.81	0	0.97	0.093	0.83
SMOTE_Kmeans_Boruta_LR	0.75	0.84	0.5	0.9	0.5	0.91
SMOTE_Kmeans_Boruta_NB	0.75	0.86	0.67	0.9	0.56	0.93
SMOTE_Kmeans_Boruta_KNN	0.75	0.67	0.67	0.68	0.28	0.91
SMOTE_Kmeans_Boruta_GB	0.75	0.19	1	0.033	0.16	1
SMOTE_Kmeans_RFE_GB	0.75	0.3	0.84	0.19	0.16	0.86
SMOTE_Kmeans_RFE_XGB	0.75	0.81	0.5	0.87	0.43	0.9
SMOTE_Local-Active-Contour_MRMR_LR	0.75	0.76	0.67	0.78	0.36	0.92
Orginal_Fuzzy-C-means_Boruta_LDA	0.74	0.84	0	1	0.9	0.84
Orginal_Fuzzy-C-means_Boruta_XGB	0.74	0.89	0.5	0.97	0.74	0.91
Orginal_Fuzzy-C-means_Boruta_RF	0.74	0.84	0	1	0.9	0.84
Orginal_Iterative-Threshold-30_MRMR_NB	0.74	0.38	0.83	0.29	0.19	0.9
Orginal_Iterative-Threshold-35_MRMR_DT	0.74	0.81	0	0.97	0.27	0.84
Orginal_Iterative-Threshold-40_MRMR_GB	0.74	0.81	0	0.97	0.011	0.83
Orginal_Iterative-Threshold-50_Boruta_NB	0.74	0.89	0.67	0.94	0.68	0.94
Orginal_Iterative-Threshold-50_Boruta_GB	0.74	0.84	0	1	0.37	0.84
Orginal_Iterative-Threshold-50_Boruta_DT	0.74	0.81	0	0.97	0.17	0.83
Orginal_Iterative-Threshold-60_Boruta_SVM	0.74	0.84	0	1	0.73	0.84
Orginal_Iterative-Threshold-70_MRMR_MLP	0.74	0.84	0	1	0.68	0.84
Orginal_Iterative-Threshold-70_RFE_GB	0.74	0.84	0	1	0.32	0.84
Orginal_Iterative-Threshold-80_RFE_MLP	0.74	0.84	0	1	0.96	0.84
Orginal_Kmeans_MRMR_MLP	0.74	0.89	0.5	0.97	0.74	0.91
Orginal_Local-Active-Contour_Boruta_LDA	0.74	0.86	0.33	0.97	0.67	0.88
Orginal_Local-Active-Contour_Boruta_RF	0.74	0.86	0.33	0.97	0.67	0.88
Orginal_Local-Active-Contour_MRMR_LR	0.74	0.81	0	0.97	0.36	0.83
Orginal_Manual_MRMR_NB	0.74	0.84	0	1	0.1	0.84
SMOTE_Fuzzy-C-means_Boruta_XGB	0.74	0.46	0.85	0.39	0.21	0.93
SMOTE_Iterative-Threshold-30_MRMR_LR	0.74	0.63	0.68	0.62	0.25	0.91
SMOTE_Iterative-Threshold-35_Boruta_LR	0.74	0.78	0.51	0.84	0.38	0.9
SMOTE_Iterative-Threshold-35_RFE_LR	0.74	0.7	0.68	0.71	0.31	0.92
SMOTE_Iterative-Threshold-50_Boruta_NB	0.74	0.81	0.67	0.84	0.44	0.93

SMOTE_Iterative-Threshold-50_Boruta_MLP	0.74	0.16	1	0	0.16	NA
SMOTE_Iterative-Threshold-60_Boruta_LR	0.74	0.86	0.66	0.9	0.56	0.93
SMOTE_Iterative-Threshold-70_Boruta_GB	0.74	0.27	0.83	0.16	0.16	0.83
SMOTE_Iterative-Threshold-70_RFE_GB	0.74	0.62	0.83	0.58	0.28	0.95
SMOTE_Iterative-Threshold-70_RFE_MLP	0.74	0.79	0.83	0.78	0.42	0.96
SMOTE_Kmeans_Boruta_MLP	0.74	0.33	0.83	0.23	0.17	0.88
SMOTE_Kmeans_MRMR_NB	0.74	0.29	1	0.16	0.19	1
SMOTE_Local-Active-Contour_Boruta_LR	0.74	0.78	0.67	0.81	0.4	0.93
SMOTE_Local-Active-Contour_MRMR_SVM	0.74	0.7	0.17	0.81	0.15	0.84
SMOTE_Manual_RFE_GB	0.74	0.16	1	0	0.16	NA
SMOTE_Watershed_RFE_XGB	0.74	0.54	0.83	0.48	0.23	0.94
Orginal_Fuzzy-C-means_Boruta_DT	0.73	0.89	0.5	0.97	0.74	0.91
Orginal_Iterative-Threshold-35_Boruta_KNN	0.73	0.79	0	0.94	0.039	0.83
Orginal_Iterative-Threshold-35_Boruta_XGB	0.73	0.84	0	1	0.19	0.84
Orginal_Iterative-Threshold-35_RFE_LR	0.73	0.76	0	0.9	0	0.83
Orginal_Iterative-Threshold-35_RFE_DT	0.73	0.84	0	1	0.35	0.84
Orginal_Iterative-Threshold-35_RFE_MLP	0.73	0.84	0	1	0.083	0.84
Orginal_Iterative-Threshold-45_RFE_DT	0.73	0.84	0.16	0.97	0.48	0.86
Orginal_Iterative-Threshold-50_RFE_KNN	0.73	0.84	0	1	0	0.84
Orginal_Iterative-Threshold-60_RFE_LDA	0.73	0.84	0	1	0.71	0.84
Orginal_Iterative-Threshold-60_RFE_SVM	0.73	0.84	0	1	0.26	0.84
Orginal_Iterative-Threshold-60_RFE_RF	0.73	0.84	0	1	0.71	0.84
Orginal_Iterative-Threshold-70_Boruta_MLP	0.73	0.84	0	1	0.72	0.84
Orginal_Iterative-Threshold-80_MRMR_XGB	0.73	0.84	0	1	0.26	0.84
Orginal_Iterative-Threshold-80_RFE_KNN	0.73	0.84	0	1	0.082	0.84
Orginal_Kmeans_MRMR_GB	0.73	0.87	0.33	0.97	0.64	0.88
SMOTE_Fuzzy-C-means_RFE_LDA	0.73	0.84	0.33	0.94	0.5	0.88
SMOTE_Fuzzy-C-means_RFE_RF	0.73	0.84	0.33	0.94	0.5	0.88
SMOTE_Iterative-Threshold-30_Boruta_GB	0.73	0.24	1	0.096	0.18	1
SMOTE_Iterative-Threshold-35_MRMR_MLP	0.73	0.51	0.83	0.45	0.23	0.93
SMOTE_Iterative-Threshold-35_RFE_XGB	0.73	0.75	0.33	0.84	0.29	0.86
SMOTE_Iterative-Threshold-70_RFE_LR	0.73	0.76	0.66	0.78	0.36	0.92
SMOTE_Kmeans_RFE_MLP	0.73	0.81	0.51	0.87	0.43	0.9
SMOTE_Local-Active-Contour_Boruta_NB	0.73	0.81	0.67	0.84	0.44	0.93
SMOTE_Local-Active-Contour_Boruta_GB	0.73	0.16	1	0	0.16	NA
SMOTE_Local-Active-Contour_Boruta_MLP	0.73	0.16	1	0	0.16	NA
SMOTE_Local-Active-Contour_MRMR_GB	0.73	0.19	0.84	0.063	0.15	0.68
SMOTE_Manual_Boruta_LR	0.73	0.68	0.66	0.68	0.29	0.91
SMOTE_Manual_RFE_DT	0.73	0.62	0.83	0.58	0.28	0.95
SMOTE_Region-Growing_Boruta_GB	0.73	0.19	1	0.031	0.16	1
Orginal_Fuzzy-C-means_Boruta_SVM	0.72	0.89	0.33	1	0.88	0.89
Orginal_Iterative-Threshold-30_MRMR_LR	0.72	0.81	0	0.97	0.034	0.83
Orginal_Iterative-Threshold-35_Boruta_DT	0.72	0.78	0	0.94	0.062	0.83
Orginal_Iterative-Threshold-35_MRMR_LR	0.72	0.81	0	0.97	0.24	0.83
Orginal_Iterative-Threshold-35_MRMR_NB	0.72	0.35	1	0.22	0.2	1
Orginal_Iterative-Threshold-50_MRMR_DT	0.72	0.83	0	1	0.33	0.83

Orginal_Iterative-Threshold-70_MRMR_GB	0.72	0.79	0	0.94	0.12	0.83
Orginal_Kmeans_Boruta_LDA	0.72	0.87	0.33	0.97	0.63	0.88
Orginal_Kmeans_Boruta_KNN	0.72	0.81	0.33	0.91	0.4	0.88
Orginal_Kmeans_Boruta_RF	0.72	0.87	0.33	0.97	0.63	0.88
Orginal_Kmeans_RFE_SVM	0.72	0.84	0	1	0.64	0.84
Orginal_Local-Active-Contour_Boruta_LR	0.72	0.81	0	0.97	0.37	0.83
Orginal_Local-Active-Contour_Boruta_GB	0.72	0.81	0	0.97	0.37	0.83
Orginal_Local-Active-Contour_Boruta_MLP	0.72	0.84	0	1	0.37	0.84
Orginal_Local-Active-Contour_MRMR_LDA	0.72	0.84	0	1	0.33	0.84
Orginal_Local-Active-Contour_MRMR_RF	0.72	0.84	0	1	0.33	0.84
Orginal_Local-Active-Contour_RFE_GB	0.72	0.87	0.16	1	0.66	0.86
Orginal_Manual_MRMR_KNN	0.72	0.84	0	1	0.01	0.84
SMOTE_Fuzzy-C-means_RFE_GB	0.72	0.29	1	0.16	0.18	1
SMOTE_Iterative-Threshold-60_RFE_NB	0.72	0.65	0.83	0.61	0.29	0.95
SMOTE_Iterative-Threshold-70_MRMR_GB	0.72	0.63	0.67	0.62	0.25	0.91
SMOTE_Iterative-Threshold-80_Boruta_MLP	0.72	0.51	1	0.41	0.25	1
SMOTE_Kmeans_MRMR_KNN	0.72	0.6	0.49	0.61	0.2	0.86
SMOTE_Local-Active-Contour_MRMR_KNN	0.72	0.7	0.65	0.71	0.3	0.91
SMOTE_Local-Active-Contour_MRMR_XGB	0.72	0.68	0.67	0.68	0.28	0.91
SMOTE_Local-Active-Contour_RFE_SVM	0.72	0.76	0	0.91	0.012	0.82
SMOTE_Manual_MRMR_XGB	0.72	0.65	0.83	0.61	0.29	0.95
SMOTE_Manual_RFE_KNN	0.72	0.65	0.49	0.68	0.23	0.87
SMOTE_Region-Growing_Boruta_LDA	0.72	0.76	0.51	0.8	0.33	0.89
SMOTE_Region-Growing_Boruta_RF	0.72	0.76	0.51	0.8	0.33	0.89
Orginal_Fuzzy-C-means_RFE_SVM	0.71	0.84	0	1	0.22	0.84
Orginal_Iterative-Threshold-45_Boruta_SVM	0.71	0.84	0	1	0.28	0.84
Orginal_Iterative-Threshold-50_Boruta_XGB	0.71	0.84	0	1	0.17	0.84
Orginal_Kmeans_RFE_KNN	0.71	0.84	0.49	0.9	0.49	0.9
Orginal_Manual_MRMR_LDA	0.71	0.84	0	1	0.72	0.84
Orginal_Manual_MRMR_XGB	0.71	0.81	0.17	0.94	0.34	0.86
Orginal_Manual_MRMR_RF	0.71	0.84	0	1	0.72	0.84
Orginal_Manual_RFE_LR	0.71	0.81	0.34	0.9	0.4	0.87
Orginal_Manual_RFE_LDA	0.71	0.84	0	1	0.67	0.84
Orginal_Manual_RFE_RF	0.71	0.84	0	1	0.67	0.84
Orginal_Watershed_MRMR_LR	0.71	0.81	0.16	0.93	0.35	0.85
SMOTE_Iterative-Threshold-35_Boruta_KNN	0.71	0.7	0.67	0.71	0.31	0.92
SMOTE_Iterative-Threshold-60_Boruta_MLP	0.71	0.49	0.84	0.42	0.22	0.93
SMOTE_Iterative-Threshold-70_MRMR_KNN	0.71	0.73	0.17	0.84	0.17	0.84
SMOTE_Manual_RFE_LDA	0.71	0.71	0.51	0.75	0.28	0.89
SMOTE_Manual_RFE_RF	0.71	0.71	0.51	0.75	0.28	0.89
Orginal_Iterative-Threshold-35_Boruta_LR	0.7	0.84	0.17	0.97	0.47	0.86
Orginal_Iterative-Threshold-35_MRMR_MLP	0.7	0.84	0	1	0.31	0.84
Orginal_Iterative-Threshold-40_MRMR_NB	0.7	0.7	0.5	0.74	0.27	0.88
Orginal_Iterative-Threshold-45_MRMR_DT	0.7	0.81	0	0.97	0.15	0.83
Orginal_Iterative-Threshold-80_Boruta_XGB	0.7	0.84	0	1	0.025	0.84
Orginal_Local-Active-Contour_Boruta_XGB	0.7	0.84	0	1	0.45	0.84

Orginal_Local-Active-Contour_RFE_DT	0.7	0.81	0.49	0.87	0.41	0.9
Orginal_Manual_RFE_MLP	0.7	0.84	0.17	0.97	0.45	0.86
Orginal_Region-Growing_Boruta_GB	0.7	0.84	0	1	0.29	0.84
Orginal_Region-Growing_MRMR_LDA	0.7	0.84	0	1	0.12	0.84
Orginal_Region-Growing_MRMR_RF	0.7	0.84	0	1	0.12	0.84
Orginal_Watershed_MRMR_DT	0.7	0.76	0	0.9	0.02	0.82
Orginal_Watershed_MRMR_SVM	0.7	0.84	0	1	0.094	0.84
SMOTE_Fuzzy-C-means_MRMR_DT	0.7	0.68	0.5	0.71	0.25	0.88
SMOTE_Iterative-Threshold-35_Boruta_DT	0.7	0.43	0.84	0.35	0.2	0.92
SMOTE_Iterative-Threshold-50_Boruta_KNN	0.7	0.7	0.51	0.74	0.27	0.89
SMOTE_Local-Active-Contour_MRMR_DT	0.7	0.62	0.84	0.58	0.27	0.95
SMOTE_Manual_Boruta_SVM	0.7	0.6	0.84	0.55	0.26	0.94
SMOTE_Manual_MRMR_NB	0.7	0.84	0	1	0.86	0.84
SMOTE_Manual_MRMR_KNN	0.7	0.73	0.67	0.74	0.33	0.92
SMOTE_Region-Growing_Boruta_XGB	0.7	0.78	0.34	0.87	0.33	0.87
SMOTE_Region-Growing_RFE_DT	0.7	0.67	0.5	0.71	0.25	0.88
SMOTE_Watershed_Boruta_DT	0.7	0.62	0.16	0.71	0.1	0.81
SMOTE_Watershed_RFE_LDA	0.7	0.73	0.5	0.77	0.3	0.89
SMOTE_Watershed_RFE_RF	0.7	0.73	0.5	0.77	0.3	0.89
Orginal_Fuzzy-C-means_MRMR_XGB	0.69	0.84	0	1	0.88	0.84
Orginal_Iterative-Threshold-30_Boruta_XGB	0.69	0.81	0	0.97	0.085	0.83
Orginal_Iterative-Threshold-35_MRMR_GB	0.69	0.84	0	1	0.1	0.84
Orginal_Iterative-Threshold-70_MRMR_LR	0.69	0.81	0.17	0.94	0.32	0.86
Orginal_Iterative-Threshold-80_MRMR_LDA	0.69	0.87	0.17	1	0.67	0.86
Orginal_Iterative-Threshold-80_MRMR_RF	0.69	0.87	0.17	1	0.67	0.86
Orginal_Kmeans_MRMR_KNN	0.69	0.78	0.33	0.87	0.33	0.87
Orginal_Manual_Boruta_XGB	0.69	0.84	0	1	0.003	0.84
Orginal_Manual_MRMR_MLP	0.69	0.81	0.16	0.93	0.34	0.85
Orginal_Region-Growing_Boruta_MLP	0.69	0.84	0	1	0.26	0.84
Orginal_Region-Growing_MRMR_XGB	0.69	0.84	0	1	0.33	0.84
Orginal_Region-Growing_MRMR_DT	0.69	0.76	0	0.9	0.029	0.82
Orginal_Region-Growing_RFE_LDA	0.69	0.83	0	1	0.086	0.83
Orginal_Region-Growing_RFE_RF	0.69	0.83	0	1	0.086	0.83
Orginal_Watershed_MRMR_GB	0.69	0.86	0.16	1	0.73	0.86
Orginal_Watershed_RFE_XGB	0.69	0.79	0	0.94	0.086	0.83
SMOTE_Fuzzy-C-means_MRMR_KNN	0.69	0.76	0.67	0.78	0.37	0.92
SMOTE_Iterative-Threshold-35_RFE_LDA	0.69	0.62	0.66	0.61	0.25	0.9
SMOTE_Iterative-Threshold-35_RFE_RF	0.69	0.62	0.66	0.61	0.25	0.9
SMOTE_Iterative-Threshold-40_MRMR_LDA	0.69	0.68	0.49	0.71	0.25	0.88
SMOTE_Iterative-Threshold-40_MRMR_RF	0.69	0.68	0.49	0.71	0.25	0.88
SMOTE_Iterative-Threshold-50_Boruta_LDA	0.69	0.49	0.67	0.45	0.19	0.88
SMOTE_Iterative-Threshold-50_Boruta_RF	0.69	0.49	0.67	0.45	0.19	0.88
SMOTE_Iterative-Threshold-50_MRMR_KNN	0.69	0.65	0.5	0.68	0.24	0.87
SMOTE_Iterative-Threshold-80_RFE_SVM	0.69	0.78	0.5	0.84	0.38	0.9
SMOTE_Kmeans_MRMR_DT	0.69	0.59	0.83	0.55	0.26	0.94
SMOTE_Kmeans_RFE_LDA	0.69	0.78	0.5	0.84	0.37	0.9

SMOTE_Kmeans_RFE_RF	0.69	0.78	0.5	0.84	0.37	0.9
SMOTE_Local-Active-Contour_Boruta_LDA	0.69	0.35	0.84	0.26	0.18	0.89
SMOTE_Local-Active-Contour_Boruta_RF	0.69	0.35	0.84	0.26	0.18	0.89
SMOTE_Local-Active-Contour_MRMR_MLP	0.69	0.68	0.67	0.68	0.28	0.91
SMOTE_Manual_MRMR_MLP	0.69	0.65	0.67	0.65	0.26	0.91
SMOTE_Watershed_MRMR_LR	0.69	0.78	0.66	0.8	0.39	0.93
SMOTE_Watershed_MRMR_XGB	0.69	0.76	0.5	0.81	0.33	0.9
Orginal_Fuzzy-C-means_RFE_KNN	0.68	0.84	0.17	0.97	0.44	0.86
Orginal_Iterative-Threshold-30_RFE_NB	0.68	0.51	0.83	0.45	0.23	0.93
Orginal_Iterative-Threshold-35_MRMR_LDA	0.68	0.84	0	1	0.077	0.84
Orginal_Iterative-Threshold-35_MRMR_RF	0.68	0.84	0	1	0.077	0.84
Orginal_Iterative-Threshold-35_RFE_GB	0.68	0.81	0	0.97	0.31	0.84
Orginal_Iterative-Threshold-40_MRMR_LDA	0.68	0.81	0	0.97	0.094	0.83
Orginal_Iterative-Threshold-40_MRMR_RF	0.68	0.81	0	0.97	0.094	0.83
Orginal_Iterative-Threshold-40_RFE_GB	0.68	0.84	0	1	0.37	0.84
Orginal_Iterative-Threshold-40_RFE_MLP	0.68	0.84	0	1	0.37	0.84
Orginal_Iterative-Threshold-45_MRMR_SVM	0.68	0.84	0	1	0.7	0.84
Orginal_Iterative-Threshold-60_Boruta_DT	0.68	0.75	0	0.9	0.027	0.82
Orginal_Iterative-Threshold-60_MRMR_KNN	0.68	0.81	0	0.97	0.005	0.83
Orginal_Iterative-Threshold-70_RFE_LR	0.68	0.81	0.16	0.94	0.32	0.85
Orginal_Manual_MRMR_LR	0.68	0.81	0.16	0.93	0.35	0.85
Orginal_Manual_MRMR_GB	0.68	0.81	0.16	0.93	0.35	0.85
Orginal_Manual_MRMR_SVM	0.68	0.84	0	1	0.22	0.84
Orginal_Watershed_MRMR_MLP	0.68	0.89	0.49	0.97	0.72	0.91
SMOTE_Iterative-Threshold-40_MRMR_NB	0.68	0.54	0.83	0.49	0.24	0.94
SMOTE_Iterative-Threshold-45_Boruta_LR	0.68	0.78	0.51	0.84	0.38	0.9
SMOTE_Iterative-Threshold-45_Boruta_LDA	0.68	0.38	0.84	0.29	0.19	0.9
SMOTE_Iterative-Threshold-45_Boruta_RF	0.68	0.38	0.84	0.29	0.19	0.9
SMOTE_Iterative-Threshold-50_RFE_KNN	0.68	0.73	0.33	0.81	0.25	0.86
SMOTE_Iterative-Threshold-60_Boruta_NB	0.68	0.67	0.34	0.74	0.2	0.85
SMOTE_Iterative-Threshold-60_RFE_KNN	0.68	0.76	0.67	0.77	0.36	0.92
SMOTE_Iterative-Threshold-70_Boruta_LR	0.68	0.76	0.5	0.81	0.34	0.89
SMOTE_Local-Active-Contour_RFE_GB	0.68	0.25	0.84	0.13	0.16	0.81
SMOTE_Region-Growing_RFE_LDA	0.68	0.73	0.67	0.74	0.33	0.92
SMOTE_Region-Growing_RFE_RF	0.68	0.73	0.67	0.74	0.33	0.92
SMOTE_Region-Growing_RFE_MLP	0.68	0.84	0.67	0.87	0.49	0.93
SMOTE_Watershed_MRMR_LDA	0.68	0.7	0.34	0.77	0.22	0.86
SMOTE_Watershed_MRMR_RF	0.68	0.7	0.34	0.77	0.22	0.86
Orginal_Iterative-Threshold-35_RFE_LDA	0.67	0.84	0	1	0	0.84
Orginal_Iterative-Threshold-35_RFE_RF	0.67	0.84	0	1	0	0.84
Orginal_Iterative-Threshold-40_MRMR_MLP	0.67	0.84	0	1	0.12	0.84
Orginal_Iterative-Threshold-60_MRMR_XGB	0.67	0.83	0	1	0.36	0.83
Orginal_Iterative-Threshold-60_MRMR_DT	0.67	0.8	0.25	0.91	0.35	0.86
Orginal_Iterative-Threshold-80_MRMR_SVM	0.67	0.87	0.17	1	0.87	0.86
Orginal_Watershed_RFE_NB	0.67	0.84	0	1	0.9	0.84
SMOTE_Iterative-Threshold-35_MRMR_KNN	0.67	0.65	0.83	0.61	0.29	0.95

SMOTE_Iterative-Threshold-40_MRMR_MLP	0.67	0.65	0.66	0.64	0.26	0.91
SMOTE_Iterative-Threshold-60_MRMR_KNN	0.67	0.7	0.34	0.77	0.22	0.86
SMOTE_Iterative-Threshold-70_RFE_NB	0.67	0.81	0.66	0.84	0.44	0.93
SMOTE_Iterative-Threshold-70_RFE_XGB	0.67	0.81	0.33	0.91	0.4	0.88
SMOTE_Iterative-Threshold-80_RFE_LR	0.67	0.7	0.51	0.74	0.27	0.88
SMOTE_Kmeans_RFE_LR	0.67	0.7	0.5	0.74	0.27	0.89
SMOTE_Local-Active-Contour_Boruta_XGB	0.67	0.41	0.84	0.32	0.19	0.91
SMOTE_Manual_MRMR_LR	0.67	0.73	0.67	0.74	0.33	0.92
SMOTE_Manual_MRMR_GB	0.67	0.16	1	0	0.16	NA
Orginal_Iterative-Threshold-30_RFE_LDA	0.66	0.86	0.16	1	0.64	0.86
Orginal_Iterative-Threshold-30_RFE_RF	0.66	0.86	0.16	1	0.64	0.86
Orginal_Iterative-Threshold-40_MRMR_XGB	0.66	0.84	0	1	0.68	0.84
Orginal_Iterative-Threshold-70_Boruta_LDA	0.66	0.84	0	1	0.65	0.84
Orginal_Iterative-Threshold-70_Boruta_GB	0.66	0.84	0	1	0.32	0.84
Orginal_Iterative-Threshold-70_Boruta_RF	0.66	0.84	0	1	0.65	0.84
Orginal_Iterative-Threshold-70_RFE_SVM	0.66	0.84	0	1	0.86	0.84
Orginal_Iterative-Threshold-80_RFE_LR	0.66	0.84	0	1	0.034	0.84
Orginal_Kmeans_MRMR_LDA	0.66	0.84	0	1	0.31	0.84
Orginal_Kmeans_MRMR_RF	0.66	0.84	0	1	0.31	0.84
Orginal_Local-Active-Contour_MRMR_KNN	0.66	0.78	0.49	0.84	0.37	0.9
SMOTE_Iterative-Threshold-40_Boruta_XGB	0.66	0.6	0.51	0.61	0.21	0.86
SMOTE_Iterative-Threshold-60_Boruta_DT	0.66	0.54	0.66	0.52	0.21	0.89
SMOTE_Iterative-Threshold-60_RFE_LDA	0.66	0.76	0.33	0.84	0.29	0.87
SMOTE_Iterative-Threshold-60_RFE_RF	0.66	0.76	0.33	0.84	0.29	0.87
SMOTE_Iterative-Threshold-70_MRMR_LR	0.66	0.79	0.5	0.84	0.38	0.9
SMOTE_Iterative-Threshold-80_MRMR_SVM	0.66	0.84	0	1	0.69	0.84
SMOTE_Local-Active-Contour_RFE_XGB	0.66	0.79	0.34	0.87	0.34	0.87
SMOTE_Watershed_Boruta_GB	0.66	0.19	1	0.031	0.17	1
SMOTE_Watershed_MRMR_MLP	0.66	0.68	0.83	0.65	0.31	0.95
Orginal_Iterative-Threshold-40_Boruta_XGB	0.65	0.84	0	1	0.006	0.84
Orginal_Iterative-Threshold-40_RFE_SVM	0.65	0.81	0.17	0.94	0.35	0.85
Orginal_Iterative-Threshold-70_Boruta_DT	0.65	0.81	0.17	0.93	0.31	0.85
Orginal_Iterative-Threshold-70_MRMR_XGB	0.65	0.87	0.17	1	0.64	0.86
Orginal_Iterative-Threshold-70_RFE_KNN	0.65	0.84	0	1	0.009	0.84
Orginal_Kmeans_Boruta_SVM	0.65	0.84	0	1	0.31	0.84
Orginal_Region-Growing_Boruta_LDA	0.65	0.84	0	1	0.25	0.84
Orginal_Region-Growing_Boruta_RF	0.65	0.84	0	1	0.25	0.84
SMOTE_Iterative-Threshold-40_MRMR_SVM	0.65	0.84	0	1	0.09	0.84
SMOTE_Iterative-Threshold-45_RFE_XGB	0.65	0.7	0.17	0.81	0.14	0.83
SMOTE_Manual_MRMR_LDA	0.65	0.37	0.83	0.29	0.18	0.9
SMOTE_Manual_MRMR_RF	0.65	0.37	0.83	0.29	0.18	0.9
Orginal_Iterative-Threshold-40_RFE_LR	0.64	0.84	0.17	0.97	0.56	0.86
Orginal_Iterative-Threshold-80_Boruta_DT	0.64	0.75	0	0.9	0.016	0.82
Orginal_Local-Active-Contour_MRMR_SVM	0.64	0.84	0	1	0.002	0.84
Orginal_Manual_Boruta_LR	0.64	0.84	0.17	0.97	0.45	0.86
SMOTE_Iterative-Threshold-30_RFE_MLP	0.64	0.54	0.68	0.52	0.22	0.89

SMOTE_Iterative-Threshold-40_RFE_GB	0.64	0.24	0.83	0.13	0.15	0.81
SMOTE_Kmeans_Boruta_LDA	0.64	0.59	0.67	0.58	0.23	0.9
SMOTE_Kmeans_Boruta_XGB	0.64	0.57	0.67	0.55	0.22	0.9
SMOTE_Kmeans_Boruta_RF	0.64	0.59	0.67	0.58	0.23	0.9
SMOTE_Manual_Boruta_MLP	0.64	0.54	0.84	0.49	0.24	0.94
SMOTE_Manual_MRMR_SVM	0.64	0.79	0	0.94	0.038	0.83
SMOTE_Manual_RFE_LR	0.64	0.65	0.67	0.65	0.27	0.91
SMOTE_Region-Growing_MRMR_KNN	0.64	0.73	0.5	0.77	0.29	0.89
SMOTE_Region-Growing_RFE_NB	0.64	0.68	0.5	0.71	0.25	0.88
SMOTE_Region-Growing_RFE_XGB	0.64	0.65	0.33	0.71	0.18	0.85
Orginal_Fuzzy-C-means_RFE_XGB	0.63	0.84	0	1	0.87	0.84
Orginal_Iterative-Threshold-30_RFE_LR	0.63	0.81	0	0.97	0.096	0.83
Orginal_Iterative-Threshold-70_RFE_LDA	0.63	0.86	0.16	1	0.74	0.86
Orginal_Iterative-Threshold-70_RFE_RF	0.63	0.86	0.16	1	0.74	0.86
Orginal_Kmeans_Boruta_XGB	0.63	0.87	0.33	0.97	0.62	0.88
Orginal_Kmeans_Boruta_DT	0.63	0.81	0.33	0.9	0.39	0.88
Orginal_Kmeans_RFE_DT	0.63	0.81	0.33	0.9	0.39	0.88
Orginal_Manual_MRMR_DT	0.63	0.81	0.52	0.87	0.43	0.9
Orginal_Watershed_MRMR_KNN	0.63	0.81	0	0.97	0.011	0.83
SMOTE_Iterative-Threshold-30_Boruta_XGB	0.63	0.68	0.33	0.74	0.21	0.85
SMOTE_Iterative-Threshold-40_Boruta_SVM	0.63	0.62	0.34	0.68	0.17	0.84
SMOTE_Kmeans_MRMR_MLP	0.63	0.49	0.66	0.45	0.19	0.88
SMOTE_Manual_Boruta_LDA	0.63	0.54	0.5	0.55	0.18	0.85
SMOTE_Manual_Boruta_RF	0.63	0.54	0.5	0.55	0.18	0.85
Orginal_Iterative-Threshold-30_Boruta_LR	0.62	0.81	0.17	0.94	0.36	0.85
Orginal_Iterative-Threshold-35_RFE_NB	0.62	0.62	0.33	0.67	0.16	0.84
Orginal_Iterative-Threshold-35_RFE_KNN	0.62	0.84	0	1	0.001	0.84
Orginal_Iterative-Threshold-40_MRMR_SVM	0.62	0.84	0	1	0.023	0.84
Orginal_Iterative-Threshold-45_Boruta_LR	0.62	0.81	0	0.97	0.25	0.83
Orginal_Iterative-Threshold-70_Boruta_NB	0.62	0.81	0.33	0.9	0.4	0.87
Orginal_Iterative-Threshold-80_Boruta_MLP	0.62	0.84	0	1	0.11	0.84
Orginal_Manual_Boruta_MLP	0.62	0.81	0	0.97	0.035	0.83
Orginal_Watershed_RFE_SVM	0.62	0.84	0	1	0.008	0.84
SMOTE_Iterative-Threshold-35_Boruta_LDA	0.62	0.57	0.84	0.51	0.25	0.94
SMOTE_Iterative-Threshold-35_Boruta_RF	0.62	0.57	0.84	0.51	0.25	0.94
SMOTE_Iterative-Threshold-35_RFE_SVM	0.62	0.84	0	1	0.013	0.84
SMOTE_Iterative-Threshold-50_Boruta_DT	0.62	0.46	0.83	0.39	0.21	0.92
SMOTE_Iterative-Threshold-70_MRMR_MLP	0.62	0.79	0.17	0.9	0.26	0.85
SMOTE_Iterative-Threshold-70_RFE_LDA	0.62	0.76	0.33	0.84	0.29	0.87
SMOTE_Iterative-Threshold-70_RFE_RF	0.62	0.76	0.33	0.84	0.29	0.87
SMOTE_Kmeans_MRMR_LDA	0.62	0.59	0.49	0.61	0.2	0.86
SMOTE_Kmeans_MRMR_RF	0.62	0.59	0.49	0.61	0.2	0.86
SMOTE_Region-Growing_Boruta_MLP	0.62	0.59	0.67	0.58	0.23	0.9
Orginal_Iterative-Threshold-40_RFE_DT	0.61	0.76	0	0.9	0.026	0.82
Orginal_Iterative-Threshold-45_Boruta_DT	0.61	0.81	0.17	0.94	0.35	0.86
Orginal_Iterative-Threshold-50_MRMR_KNN	0.61	0.81	0	0.97	0.12	0.83

Orginal_Iterative-Threshold-80_RFE_NB	0.61	0.27	0.67	0.19	0.14	0.76
Orginal_Local-Active-Contour_Boruta_SVM	0.61	0.84	0	1	0.87	0.84
Orginal_Local-Active-Contour_RFE_LR	0.61	0.87	0.16	1	0.86	0.86
SMOTE_Fuzzy-C-means_Boruta_SVM	0.61	0.62	0.34	0.68	0.17	0.84
SMOTE_Iterative-Threshold-35_MRMR_SVM	0.61	0.84	0	1	0.64	0.84
SMOTE_Iterative-Threshold-40_MRMR_XGB	0.61	0.68	0.17	0.77	0.13	0.83
SMOTE_Iterative-Threshold-40_RFE_LR	0.61	0.59	0.49	0.61	0.2	0.87
SMOTE_Iterative-Threshold-80_RFE_LDA	0.61	0.76	0.35	0.84	0.3	0.87
SMOTE_Iterative-Threshold-80_RFE_RF	0.61	0.76	0.35	0.84	0.3	0.87
SMOTE_Region-Growing_RFE_KNN	0.61	0.81	0.16	0.94	0.34	0.86
SMOTE_Watershed_RFE_NB	0.61	0.68	0.5	0.71	0.25	0.88
Orginal_Fuzzy-C-means_MRMR_KNN	0.6	0.73	0.34	0.81	0.26	0.86
Orginal_Iterative-Threshold-40_Boruta_KNN	0.6	0.7	0.51	0.74	0.27	0.89
Orginal_Iterative-Threshold-40_RFE_LDA	0.6	0.84	0	1	0.54	0.84
Orginal_Iterative-Threshold-40_RFE_RF	0.6	0.84	0	1	0.54	0.84
Orginal_Iterative-Threshold-80_MRMR_NB	0.6	0.25	0.67	0.16	0.13	0.72
Orginal_Local-Active-Contour_Boruta_KNN	0.6	0.81	0.33	0.9	0.41	0.87
Orginal_Local-Active-Contour_MRMR_DT	0.6	0.68	0.5	0.71	0.25	0.88
Orginal_Region-Growing_MRMR_KNN	0.6	0.84	0	1	0.22	0.84
SMOTE_Iterative-Threshold-35_Boruta_XGB	0.6	0.51	0.84	0.45	0.23	0.94
SMOTE_Iterative-Threshold-50_MRMR_LDA	0.6	0.67	0.16	0.77	0.13	0.83
SMOTE_Iterative-Threshold-50_MRMR_RF	0.6	0.67	0.16	0.77	0.13	0.83
SMOTE_Iterative-Threshold-50_RFE_DT	0.6	0.73	0.5	0.77	0.3	0.89
SMOTE_Iterative-Threshold-70_Boruta_NB	0.6	0.71	0.5	0.75	0.28	0.89
SMOTE_Manual_MRMR_DT	0.6	0.51	0.83	0.45	0.22	0.93
SMOTE_Watershed_MRMR_DT	0.6	0.68	0.5	0.71	0.24	0.88
Orginal_Iterative-Threshold-30_MRMR_LDA	0.59	0.84	0	1	0.008	0.84
Orginal_Iterative-Threshold-30_MRMR_RF	0.59	0.84	0	1	0.008	0.84
Orginal_Iterative-Threshold-50_RFE_LDA	0.59	0.78	0	0.93	0	0.83
Orginal_Iterative-Threshold-50_RFE_RF	0.59	0.78	0	0.93	0	0.83
Orginal_Iterative-Threshold-60_RFE_KNN	0.59	0.79	0.34	0.87	0.34	0.87
Orginal_Iterative-Threshold-70_Boruta_XGB	0.59	0.84	0	1	0.64	0.84
Orginal_Iterative-Threshold-70_MRMR_NB	0.59	0.3	0.83	0.2	0.17	0.86
Orginal_Iterative-Threshold-70_RFE_DT	0.59	0.84	0.33	0.94	0.49	0.88
Orginal_Iterative-Threshold-80_RFE_LDA	0.59	0.84	0.18	0.97	0.45	0.86
Orginal_Iterative-Threshold-80_RFE_RF	0.59	0.84	0.18	0.97	0.45	0.86
Orginal_Kmeans_MRMR_SVM	0.59	0.84	0	1	0	0.84
SMOTE_Fuzzy-C-means_RFE_SVM	0.59	0.84	0	1	0.013	0.84
SMOTE_Iterative-Threshold-40_Boruta_KNN	0.59	0.62	0.51	0.65	0.22	0.87
SMOTE_Iterative-Threshold-45_Boruta_XGB	0.59	0.49	0.68	0.45	0.19	0.88
SMOTE_Iterative-Threshold-45_RFE_LDA	0.59	0.6	0.34	0.65	0.16	0.83
SMOTE_Iterative-Threshold-45_RFE_RF	0.59	0.6	0.34	0.65	0.16	0.83
SMOTE_Iterative-Threshold-70_Boruta_KNN	0.59	0.73	0.17	0.84	0.17	0.84
SMOTE_Local-Active-Contour_RFE_LR	0.59	0.65	0.51	0.68	0.24	0.88
Orginal_Iterative-Threshold-35_MRMR_XGB	0.58	0.84	0	1	0.12	0.84
Orginal_Iterative-Threshold-40_RFE_XGB	0.58	0.84	0	1	0.54	0.84

Orginal_Iterative-Threshold-45_RFE_XGB	0.58	0.84	0	1	0.091	0.84
Orginal_Region-Growing_MRMR_SVM	0.58	0.84	0	1	0.004	0.84
SMOTE_Iterative-Threshold-45_RFE_DT	0.58	0.49	0.34	0.52	0.12	0.8
SMOTE_Iterative-Threshold-60_MRMR_XGB	0.58	0.67	0.33	0.74	0.2	0.85
SMOTE_Iterative-Threshold-70_RFE_KNN	0.58	0.73	0.17	0.84	0.18	0.84
SMOTE_Iterative-Threshold-80_RFE_NB	0.58	0.27	0.68	0.19	0.14	0.76
SMOTE_Kmeans_Boruta_DT	0.58	0.59	0.67	0.58	0.23	0.9
SMOTE_Local-Active-Contour_Boruta_SVM	0.58	0.22	1	0.064	0.17	1
SMOTE_Manual_RFE_XGB	0.58	0.68	0.34	0.74	0.2	0.85
Orginal_Iterative-Threshold-35_MRMR_KNN	0.57	0.73	0.17	0.84	0.17	0.84
Orginal_Iterative-Threshold-50_Boruta_SVM	0.57	0.84	0	1	0.68	0.84
Orginal_Iterative-Threshold-70_MRMR_KNN	0.57	0.84	0	1	0.013	0.84
Orginal_Iterative-Threshold-70_RFE_XGB	0.57	0.81	0	0.97	0.24	0.83
Orginal_Local-Active-Contour_Boruta_DT	0.57	0.84	0.16	0.97	0.46	0.85
Orginal_Region-Growing_Boruta_SVM	0.57	0.81	0	0.97	0.037	0.83
SMOTE_Fuzzy-C-means_RFE_DT	0.57	0.65	0.34	0.71	0.18	0.85
SMOTE_Fuzzy-C-means_RFE_MLP	0.57	0.68	0.17	0.77	0.13	0.83
SMOTE_Iterative-Threshold-35_MRMR_LDA	0.57	0.76	0.33	0.84	0.28	0.87
SMOTE_Iterative-Threshold-35_MRMR_RF	0.57	0.76	0.33	0.84	0.28	0.87
SMOTE_Iterative-Threshold-70_Boruta_LDA	0.57	0.43	0.51	0.42	0.14	0.82
SMOTE_Iterative-Threshold-70_Boruta_RF	0.57	0.43	0.51	0.42	0.14	0.82
Orginal_Fuzzy-C-means_MRMR_LDA	0.56	0.84	0	1	0.63	0.84
Orginal_Fuzzy-C-means_MRMR_RF	0.56	0.84	0	1	0.63	0.84
Orginal_Iterative-Threshold-50_RFE_SVM	0.56	0.84	0	1	0	0.84
Orginal_Kmeans_RFE_LR	0.56	0.81	0.34	0.9	0.4	0.88
Orginal_Region-Growing_RFE_XGB	0.56	0.81	0	0.97	0.009	0.83
SMOTE_Iterative-Threshold-45_MRMR_LDA	0.56	0.59	0.33	0.64	0.15	0.83
SMOTE_Iterative-Threshold-45_MRMR_XGB	0.56	0.65	0.33	0.71	0.18	0.85
SMOTE_Iterative-Threshold-45_MRMR_RF	0.56	0.59	0.33	0.64	0.15	0.83
SMOTE_Iterative-Threshold-50_Boruta_LR	0.56	0.68	0.51	0.71	0.25	0.88
SMOTE_Iterative-Threshold-70_RFE_DT	0.56	0.79	0.5	0.84	0.38	0.9
SMOTE_Region-Growing_Boruta_KNN	0.56	0.73	0.17	0.84	0.17	0.84
SMOTE_Iterative-Threshold-40_RFE_MLP	0.55	0.35	0.66	0.29	0.15	0.82
SMOTE_Iterative-Threshold-45_RFE_SVM	0.55	0.81	0	0.97	0.24	0.83
SMOTE_Iterative-Threshold-50_MRMR_SVM	0.55	0.65	0	0.77	0	0.8
SMOTE_Iterative-Threshold-70_MRMR_SVM	0.55	0.81	0	0.97	0.005	0.84
SMOTE_Manual_RFE_SVM	0.55	0.84	0	1	0	0.84
Orginal_Fuzzy-C-means_MRMR_SVM	0.54	0.78	0	0.93	0.002	0.83
Orginal_Iterative-Threshold-35_RFE_XGB	0.54	0.82	0	0.97	0.032	0.84
Orginal_Iterative-Threshold-60_MRMR_SVM	0.54	0.83	0	1	0.011	0.83
Orginal_Iterative-Threshold-70_Boruta_LR	0.54	0.81	0	0.97	0.23	0.83
Orginal_Region-Growing_RFE_SVM	0.54	0.83	0	1	0	0.83
SMOTE_Iterative-Threshold-30_Boruta_LR	0.54	0.78	0.33	0.87	0.33	0.87
SMOTE_Iterative-Threshold-45_RFE_KNN	0.54	0.81	0.15	0.94	0.31	0.85
SMOTE_Iterative-Threshold-60_MRMR_SVM	0.54	0.81	0.16	0.94	0.31	0.85
SMOTE_Kmeans_MRMR_XGB	0.54	0.7	0.49	0.74	0.27	0.88

SMOTE_Watershed_Boruta_LDA	0.54	0.7	0.17	0.8	0.14	0.83
SMOTE_Watershed_Boruta_RF	0.54	0.7	0.17	0.8	0.14	0.83
Orginal_Iterative-Threshold-50_MRMR_LDA	0.53	0.83	0	1	0.073	0.83
Orginal_Iterative-Threshold-50_MRMR_RF	0.53	0.83	0	1	0.073	0.83
SMOTE_Fuzzy-C-means_MRMR_SVM	0.53	0.76	0.17	0.87	0.2	0.84
SMOTE_Iterative-Threshold-30_RFE_SVM	0.53	0.86	0.17	1	0.67	0.86
SMOTE_Iterative-Threshold-40_RFE_LDA	0.53	0.4	0.5	0.39	0.13	0.8
SMOTE_Iterative-Threshold-40_RFE_RF	0.53	0.4	0.5	0.39	0.13	0.8
SMOTE_Iterative-Threshold-60_RFE_SVM	0.53	0.84	0	1	0.64	0.84
SMOTE_Iterative-Threshold-70_MRMR_DT	0.53	0.49	0.5	0.49	0.16	0.84
SMOTE_Region-Growing_Boruta_DT	0.53	0.65	0.34	0.72	0.18	0.85
Orginal_Iterative-Threshold-50_Boruta_LR	0.52	0.81	0	0.97	0.25	0.83
Orginal_Iterative-Threshold-80_Boruta_LDA	0.52	0.81	0	0.96	0.038	0.83
Orginal_Iterative-Threshold-80_Boruta_RF	0.52	0.81	0	0.96	0.038	0.83
SMOTE_Iterative-Threshold-30_MRMR_LDA	0.52	0.7	0.18	0.8	0.16	0.84
SMOTE_Iterative-Threshold-30_MRMR_RF	0.52	0.7	0.18	0.8	0.16	0.84
SMOTE_Kmeans_Boruta_SVM	0.52	0.68	0.17	0.77	0.13	0.83
Orginal_Iterative-Threshold-30_MRMR_SVM	0.51	0.84	0	1	0.074	0.84
Orginal_Iterative-Threshold-60_RFE_XGB	0.51	0.84	0	1	0.66	0.84
Orginal_Manual_Boruta_SVM	0.51	0.84	0	1	0.009	0.84
Orginal_Manual_RFE_XGB	0.51	0.86	0.17	1	0.64	0.86
SMOTE_Iterative-Threshold-30_MRMR_KNN	0.51	0.59	0.32	0.64	0.15	0.83
SMOTE_Iterative-Threshold-40_Boruta_LDA	0.51	0.46	0.68	0.42	0.19	0.87
SMOTE_Iterative-Threshold-40_Boruta_RF	0.51	0.46	0.68	0.42	0.19	0.87
SMOTE_Iterative-Threshold-80_Boruta_NB	0.51	0.57	0.33	0.61	0.15	0.82
SMOTE_Kmeans_MRMR_SVM	0.51	0.73	0	0.87	0	0.82
Orginal_Iterative-Threshold-40_Boruta_DT	0.5	0.84	0	1	0.16	0.84
Orginal_Iterative-Threshold-40_MRMR_DT	0.5	0.84	0	1	0.17	0.84
Orginal_Iterative-Threshold-50_RFE_DT	0.5	0.84	0	1	0.18	0.84
Orginal_Iterative-Threshold-60_RFE_DT	0.5	0.84	0	1	0.17	0.84
Orginal_Iterative-Threshold-70_MRMR_DT	0.5	0.84	0	1	0.15	0.84
Orginal_Manual_Boruta_LDA	0.5	0.84	0	1	0.001	0.84
Orginal_Manual_Boruta_RF	0.5	0.84	0	1	0.001	0.84
Orginal_Manual_RFE_KNN	0.5	0.81	0	0.97	0	0.83
SMOTE_Iterative-Threshold-30_MRMR_SVM	0.5	0.54	0.35	0.58	0.14	0.82
SMOTE_Iterative-Threshold-35_MRMR_XGB	0.5	0.7	0.17	0.81	0.14	0.83
SMOTE_Iterative-Threshold-40_Boruta_DT	0.5	0.16	1	0	0.16	NA
SMOTE_Iterative-Threshold-80_RFE_DT	0.5	0.76	0.34	0.84	0.29	0.87
SMOTE_Local-Active-Contour_Boruta_DT	0.5	0.16	1	0	0.16	NA
Orginal_Iterative-Threshold-80_RFE_XGB	0.49	0.84	0	1	0	0.84
SMOTE_Iterative-Threshold-30_RFE_KNN	0.49	0.7	0.17	0.81	0.15	0.83
SMOTE_Iterative-Threshold-40_RFE_DT	0.49	0.24	0.83	0.13	0.15	0.81
SMOTE_Iterative-Threshold-45_Boruta_DT	0.49	0.24	0.84	0.13	0.16	0.8
SMOTE_Iterative-Threshold-70_MRMR_NB	0.49	0.38	0.5	0.36	0.13	0.79
SMOTE_Kmeans_RFE_DT	0.49	0.59	0.33	0.64	0.15	0.83
SMOTE_Region-Growing_Boruta_SVM	0.49	0.62	0.5	0.64	0.21	0.87

SMOTE_Watershed_Boruta_MLP	0.49	0.59	0.5	0.61	0.2	0.86
Orginal_Iterative-Threshold-70_Boruta_SVM	0.48	0.84	0	1	0.01	0.84
Orginal_Region-Growing_Boruta_XGB	0.48	0.84	0	1	0.062	0.84
Orginal_Watershed_Boruta_MLP	0.48	0.84	0	1	0.24	0.84
SMOTE_Iterative-Threshold-30_MRMR_MLP	0.48	0.49	0.68	0.45	0.19	0.88
SMOTE_Iterative-Threshold-30_RFE_XGB	0.48	0.73	0.17	0.84	0.17	0.84
SMOTE_Iterative-Threshold-50_MRMR_XGB	0.48	0.59	0.16	0.68	0.091	0.81
SMOTE_Iterative-Threshold-60_RFE_DT	0.48	0.54	0	0.64	0	0.77
Orginal_Iterative-Threshold-40_RFE_KNN	0.47	0.76	0	0.91	0.043	0.82
Orginal_Iterative-Threshold-45_MRMR_KNN	0.47	0.78	0	0.93	0.02	0.83
Orginal_Region-Growing_Boruta_KNN	0.47	0.81	0	0.97	0	0.83
Orginal_Watershed_MRMR_XGB	0.47	0.84	0.16	0.97	0.46	0.86
SMOTE_Iterative-Threshold-40_RFE_SVM	0.47	0.7	0.17	0.8	0.14	0.84
SMOTE_Iterative-Threshold-45_MRMR_KNN	0.47	0.68	0.17	0.77	0.13	0.83
SMOTE_Manual_Boruta_KNN	0.47	0.67	0	0.81	0.002	0.81
SMOTE_Region-Growing_Boruta_LR	0.47	0.65	0.34	0.71	0.18	0.85
SMOTE_Region-Growing_RFE_SVM	0.47	0.65	0.16	0.74	0.11	0.82
SMOTE_Watershed_RFE_KNN	0.47	0.68	0.33	0.74	0.19	0.85
Orginal_Iterative-Threshold-40_Boruta_SVM	0.46	0.84	0	1	0.005	0.84
Orginal_Iterative-Threshold-70_Boruta_KNN	0.46	0.81	0	0.97	0	0.83
Orginal_Region-Growing_RFE_KNN	0.46	0.81	0	0.97	0.006	0.83
SMOTE_Fuzzy-C-means_Boruta_MLP	0.46	0.27	0.85	0.16	0.16	0.84
SMOTE_Iterative-Threshold-35_RFE_DT	0.46	0.67	0.49	0.71	0.25	0.88
SMOTE_Iterative-Threshold-40_MRMR_KNN	0.46	0.6	0.34	0.65	0.16	0.84
SMOTE_Iterative-Threshold-40_RFE_XGB	0.46	0.48	0.5	0.48	0.15	0.83
SMOTE_Iterative-Threshold-70_MRMR_LDA	0.46	0.68	0.34	0.74	0.2	0.86
SMOTE_Iterative-Threshold-70_MRMR_RF	0.46	0.68	0.34	0.74	0.2	0.86
SMOTE_Manual_Boruta_XGB	0.46	0.62	0.34	0.68	0.17	0.84
Orginal_Iterative-Threshold-30_RFE_KNN	0.45	0.75	0	0.9	0.004	0.82
Orginal_Iterative-Threshold-45_RFE_SVM	0.45	0.84	0	1	0.075	0.84
Orginal_Watershed_RFE_KNN	0.45	0.76	0	0.9	0.013	0.83
SMOTE_Iterative-Threshold-70_Boruta_XGB	0.45	0.41	0.51	0.39	0.14	0.8
SMOTE_Iterative-Threshold-70_MRMR_XGB	0.45	0.71	0.34	0.78	0.23	0.86
SMOTE_Iterative-Threshold-80_MRMR_NB	0.45	0.24	0.66	0.16	0.13	0.71
SMOTE_Local-Active-Contour_RFE_MLP	0.45	0.66	0.34	0.72	0.19	0.85
SMOTE_Manual_Boruta_DT	0.45	0.35	0.68	0.29	0.16	0.82
SMOTE_Manual_RFE_MLP	0.45	0.65	0.17	0.75	0.11	0.82
Orginal_Iterative-Threshold-30_MRMR_KNN	0.44	0.73	0	0.87	0.002	0.82
Orginal_Region-Growing_Boruta_DT	0.44	0.81	0	0.97	0.14	0.83
Orginal_Watershed_Boruta_DT	0.44	0.57	0	0.68	0	0.78
SMOTE_Iterative-Threshold-80_Boruta_XGB	0.44	0.45	0.33	0.48	0.11	0.79
Orginal_Iterative-Threshold-45_MRMR_XGB	0.43	0.84	0	1	0.033	0.84
Orginal_Iterative-Threshold-80_Boruta_SVM	0.43	0.84	0	1	0	0.84
SMOTE_Iterative-Threshold-80_Boruta_SVM	0.43	0.51	0.33	0.55	0.13	0.81
Orginal_Iterative-Threshold-30_RFE_XGB	0.42	0.83	0	1	0.21	0.83
SMOTE_Iterative-Threshold-60_MRMR_DT	0.42	0.47	0	0.56	0	0.74

SMOTE_Iterative-Threshold-60_RFE_XGB	0.42	0.78	0.33	0.87	0.33	0.87
Orginal_Iterative-Threshold-30_MRMR_XGB	0.41	0.78	0	0.93	0	0.83
Orginal_Iterative-Threshold-35_MRMR_SVM	0.41	0.84	0	1	0	0.84
Orginal_Iterative-Threshold-70_MRMR_SVM	0.41	0.84	0	1	0.003	0.84
Orginal_Local-Active-Contour_RFE_XGB	0.41	0.87	0.16	1	0.62	0.86
Orginal_Manual_RFE_SVM	0.41	0.84	0	1	0	0.84
Orginal_Region-Growing_Boruta_LR	0.41	0.78	0.16	0.9	0.25	0.85
Orginal_Watershed_Boruta_SVM	0.41	0.84	0	1	0.005	0.84
SMOTE_Iterative-Threshold-30_RFE_LDA	0.41	0.73	0.17	0.84	0.18	0.84
SMOTE_Iterative-Threshold-30_RFE_RF	0.41	0.73	0.17	0.84	0.18	0.84
SMOTE_Iterative-Threshold-35_Boruta_SVM	0.41	0.16	1	0	0.16	NA
SMOTE_Iterative-Threshold-40_RFE_KNN	0.41	0.62	0.33	0.68	0.16	0.84
SMOTE_Iterative-Threshold-50_Boruta_SVM	0.41	0.27	0.49	0.23	0.11	0.7
Orginal_Fuzzy-C-means_RFE_DT	0.4	0.76	0	0.9	0.002	0.82
Orginal_Watershed_Boruta_XGB	0.4	0.84	0	1	0.001	0.84
SMOTE_Watershed_Boruta_LR	0.4	0.59	0.33	0.64	0.16	0.83
Orginal_Iterative-Threshold-40_MRMR_KNN	0.39	0.65	0	0.77	0	0.8
Orginal_Kmeans_MRMR_XGB	0.39	0.78	0	0.93	0.027	0.83
Orginal_Kmeans_MRMR_DT	0.39	0.78	0.33	0.87	0.33	0.87
SMOTE_Iterative-Threshold-30_MRMR_XGB	0.39	0.57	0.18	0.64	0.09	0.8
SMOTE_Iterative-Threshold-35_MRMR_DT	0.39	0.35	0.33	0.35	0.092	0.73
Orginal_Iterative-Threshold-80_Boruta_KNN	0.38	0.81	0	0.96	0.001	0.83
SMOTE_Iterative-Threshold-70_Boruta_DT	0.38	0.51	0.34	0.55	0.12	0.81
SMOTE_Iterative-Threshold-70_Boruta_SVM	0.38	0.46	0.35	0.49	0.11	0.79
SMOTE_Watershed_Boruta_KNN	0.38	0.65	0.17	0.74	0.11	0.82
Orginal_Iterative-Threshold-30_Boruta_DT	0.37	0.84	0.16	0.97	0.45	0.86
Orginal_Watershed_Boruta_LR	0.37	0.81	0	0.97	0.012	0.84
SMOTE_Iterative-Threshold-30_RFE_DT	0.37	0.73	0.17	0.84	0.17	0.84
Orginal_Watershed_Boruta_LDA	0.36	0.81	0	0.97	0.001	0.83
Orginal_Watershed_Boruta_RF	0.36	0.81	0	0.97	0.001	0.83
SMOTE_Iterative-Threshold-70_Boruta_MLP	0.36	0.33	0.34	0.33	0.086	0.72
SMOTE_Watershed_Boruta_SVM	0.36	0.7	0.17	0.8	0.14	0.83
Orginal_Iterative-Threshold-30_RFE_SVM	0.35	0.83	0	1	0.65	0.83
SMOTE_Watershed_RFE_SVM	0.35	0.44	0.18	0.49	0.059	0.76
SMOTE_Iterative-Threshold-45_Boruta_SVM	0.34	0.33	0.33	0.32	0.087	0.72
SMOTE_Iterative-Threshold-80_Boruta_LDA	0.34	0.37	0.16	0.42	0.051	0.72
SMOTE_Iterative-Threshold-80_Boruta_RF	0.34	0.37	0.16	0.42	0.051	0.72
SMOTE_Iterative-Threshold-30_Boruta_LDA	0.33	0.62	0.17	0.71	0.11	0.81
SMOTE_Iterative-Threshold-30_Boruta_RF	0.33	0.62	0.17	0.71	0.11	0.81
SMOTE_Iterative-Threshold-80_Boruta_KNN	0.33	0.59	0.16	0.68	0.093	0.81
SMOTE_Iterative-Threshold-80_MRMR_LDA	0.33	0.68	0.16	0.77	0.12	0.83
SMOTE_Iterative-Threshold-80_MRMR_XGB	0.33	0.73	0.16	0.84	0.15	0.84
SMOTE_Iterative-Threshold-80_MRMR_RF	0.33	0.68	0.16	0.77	0.12	0.83
SMOTE_Iterative-Threshold-80_RFE_XGB	0.33	0.59	0.34	0.64	0.16	0.83
Orginal_Iterative-Threshold-50_MRMR_XGB	0.32	0.78	0	0.94	0.001	0.83
Orginal_Manual_Boruta_KNN	0.32	0.84	0	1	0	0.84

Orginal_Manual_Boruta_DT	0.32	0.81	0	0.97	0.019	0.83
Orginal_Iterative-Threshold-30_RFE_DT	0.31	0.73	0.16	0.84	0.16	0.83
Orginal_Iterative-Threshold-30_MRMR_DT	0.3	0.84	0	1	0	0.84
Orginal_Iterative-Threshold-80_RFE_DT	0.3	0.81	0	0.97	0.031	0.84
SMOTE_Iterative-Threshold-50_MRMR_DT	0.3	0.62	0.33	0.68	0.17	0.84
SMOTE_Iterative-Threshold-80_Boruta_DT	0.3	0.35	0.16	0.38	0.049	0.7
SMOTE_Iterative-Threshold-30_Boruta_DT	0.28	0.65	0.17	0.74	0.12	0.82
SMOTE_Iterative-Threshold-40_MRMR_DT	0.28	0.68	0.16	0.77	0.12	0.83
Orginal_Watershed_Boruta_KNN	0.27	0.84	0	1	0	0.84
SMOTE_Iterative-Threshold-30_MRMR_DT	0.27	0.35	0.18	0.38	0.055	0.71
SMOTE_Iterative-Threshold-80_MRMR_DT	0.27	0.65	0.16	0.74	0.1	0.83
SMOTE_Watershed_Boruta_XGB	0.27	0.59	0.17	0.68	0.093	0.81
Orginal_Iterative-Threshold-50_MRMR_SVM	0.24	0.83	0	1	0.003	0.83
Orginal_Iterative-Threshold-50_RFE_XGB	0.23	0.78	0	0.93	0	0.83
SMOTE_Iterative-Threshold-45_MRMR_SVM	0.22	0.81	0	0.97	0	0.84
Orginal_Fuzzy-C-means_MRMR_DT	0.17	0.7	0	0.84	0	0.81
Orginal_Iterative-Threshold-35_RFE_SVM	0.16	0.84	0	1	0	0.84

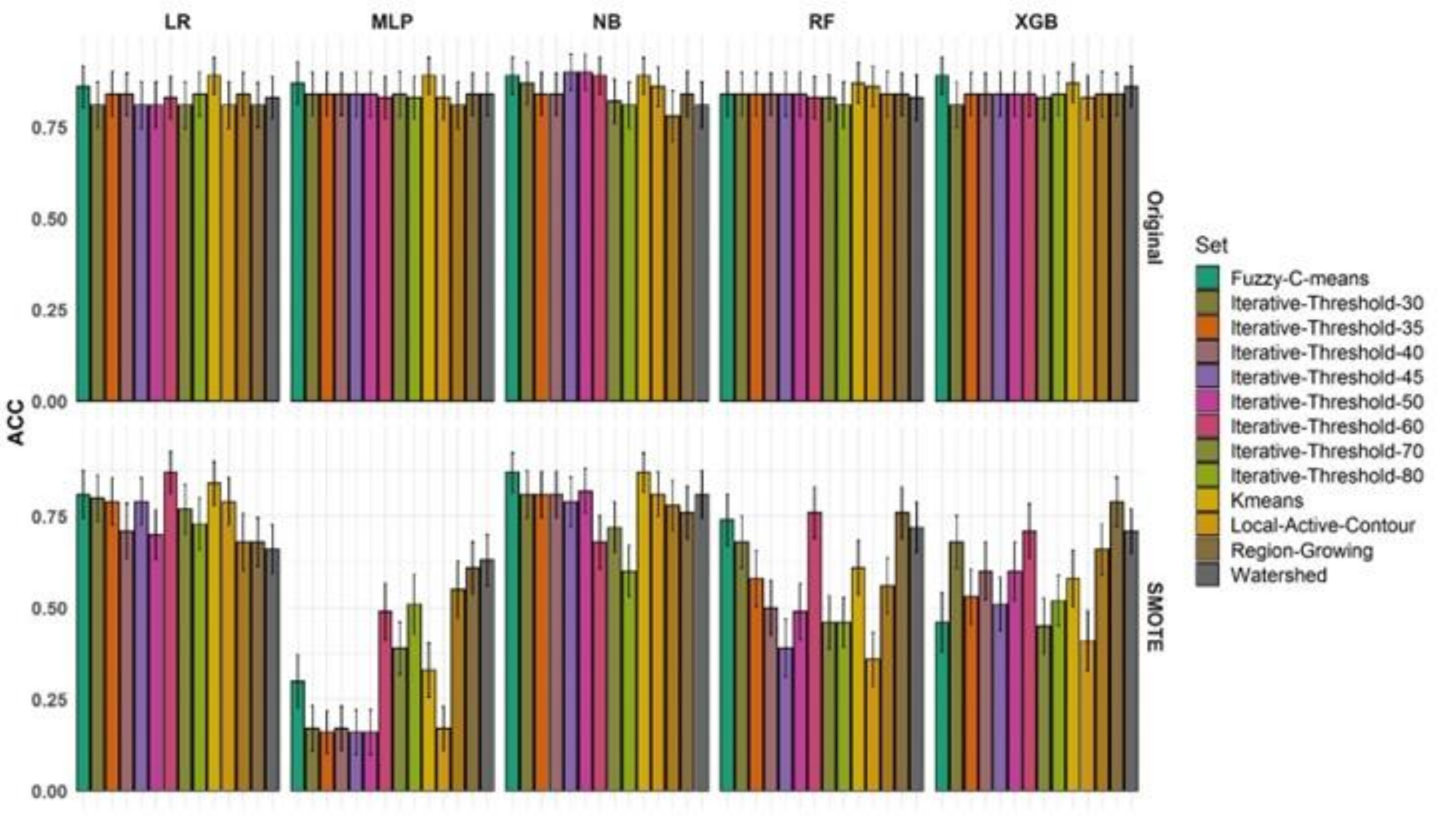
LR
MLP
NB
RF
XGB
ACC
Original
SMOTE
0.00
0.25
0.50
0.75
Set
Fuzzy-C-means
Iterative-Threshold-30
Iterative-Threshold-35
Iterative-Threshold-40
Iterative-Threshold-45
Iterative-Threshold-50
Iterative-Threshold-60
Iterative-Threshold-70
Iterative-Threshold-80
Kmeans
Local-Active-Contour
Region-Growing
Watershed

A

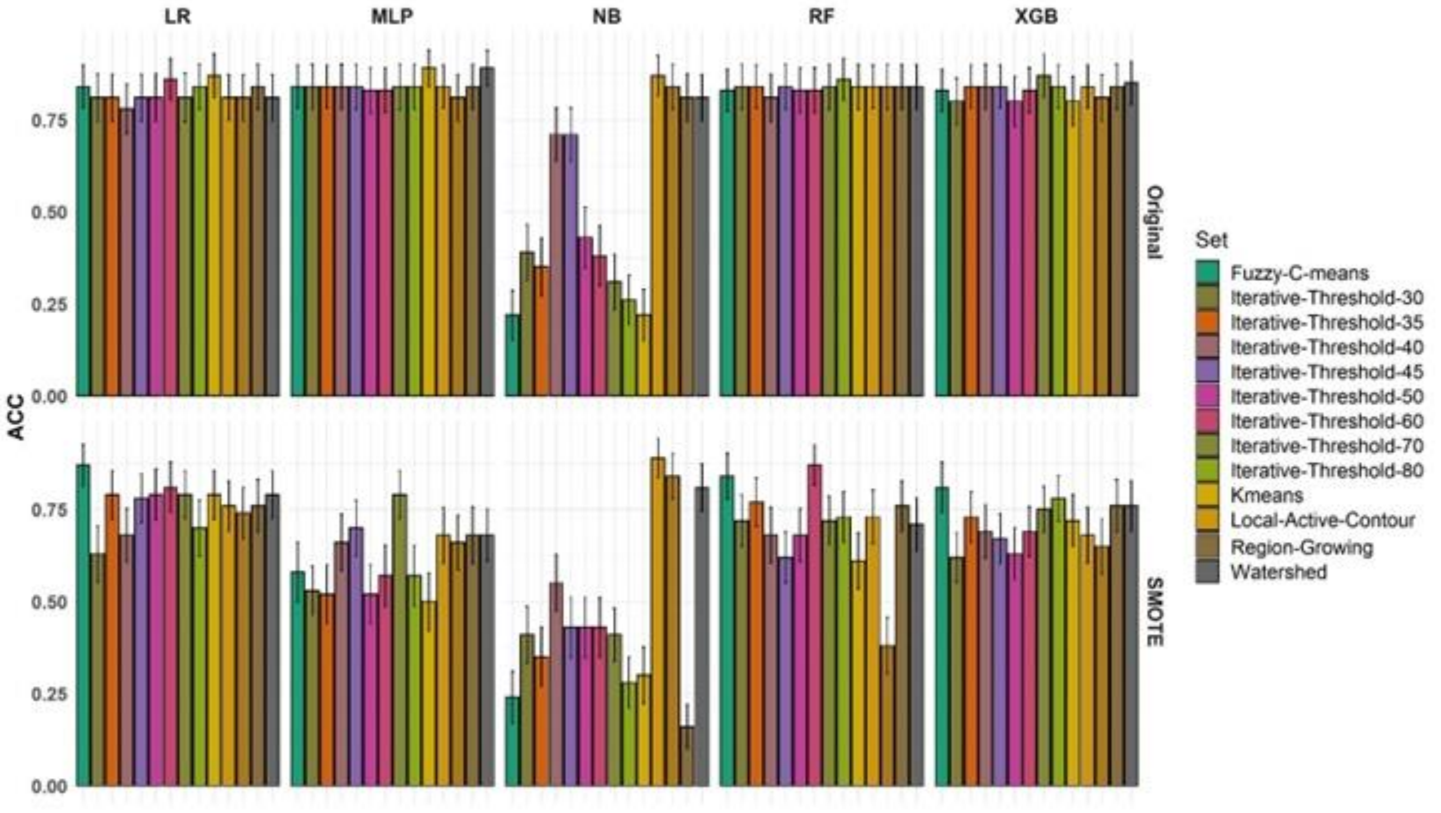
LR
MLP
NB
RF
XGB
ACC
Original
SMOTE
0.00
0.25
0.50
0.75
Set
Fuzzy-C-means
Iterative-Threshold-30
Iterative-Threshold-35
Iterative-Threshold-40
Iterative-Threshold-45
Iterative-Threshold-50
Iterative-Threshold-60
Iterative-Threshold-70
Iterative-Threshold-80
Kmeans
Local-Active-Contour
Region-Growing
Watershed

B

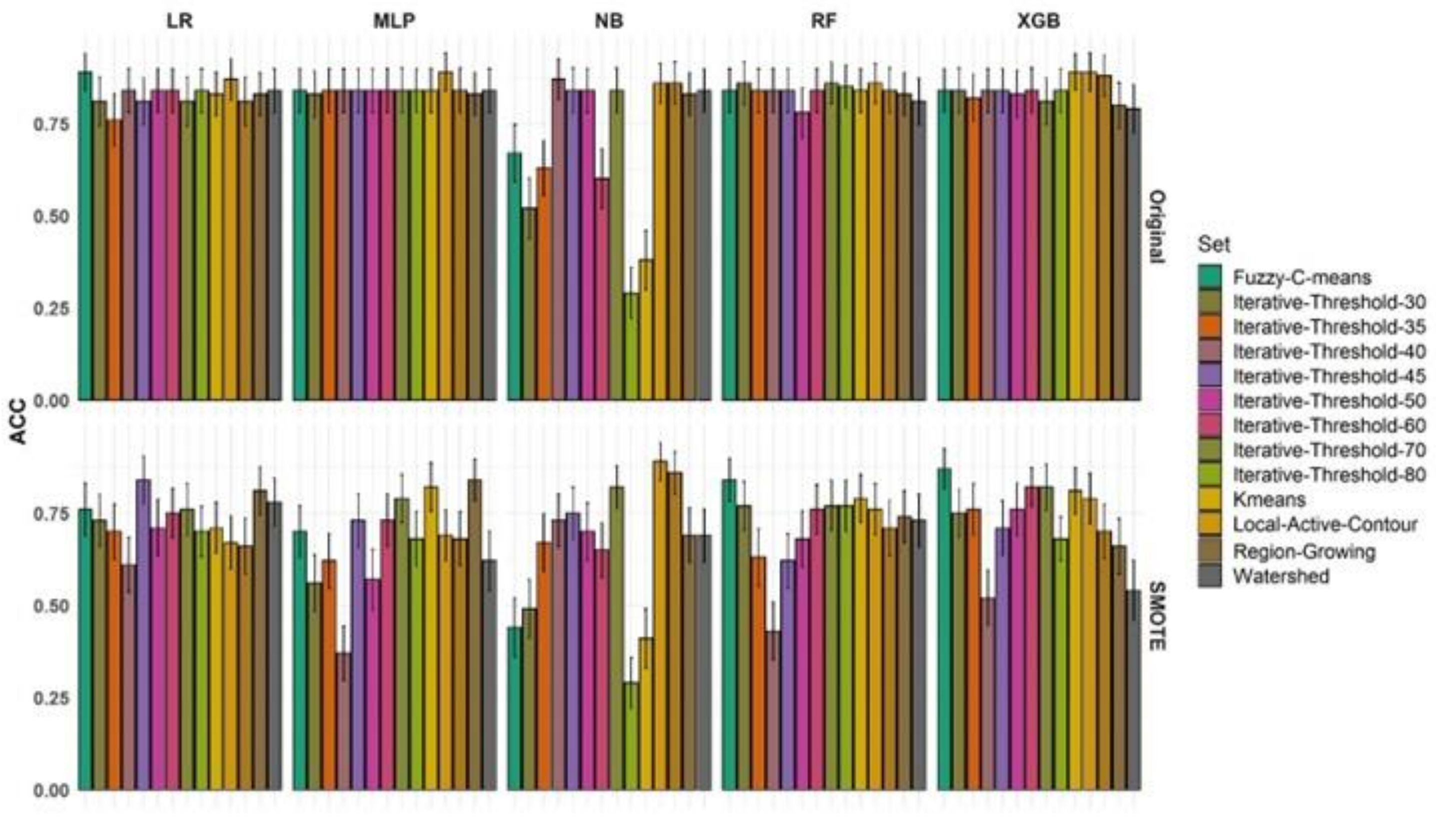

A

Figure 1. The Accuracy (ACC) belonging to Boruta (A), MRMR (B), and RFE (C) feature selectors were applied to 13 image segmentation methods, including LAC, FCM, K means, Watershed, RG, and Iterative threshold besides 5 machine learning classifiers including MLP, LR, XGB, NB, and RF. Results are reported both with conducting SMOTE (lower plots) and original without SMOTE (upper plots).

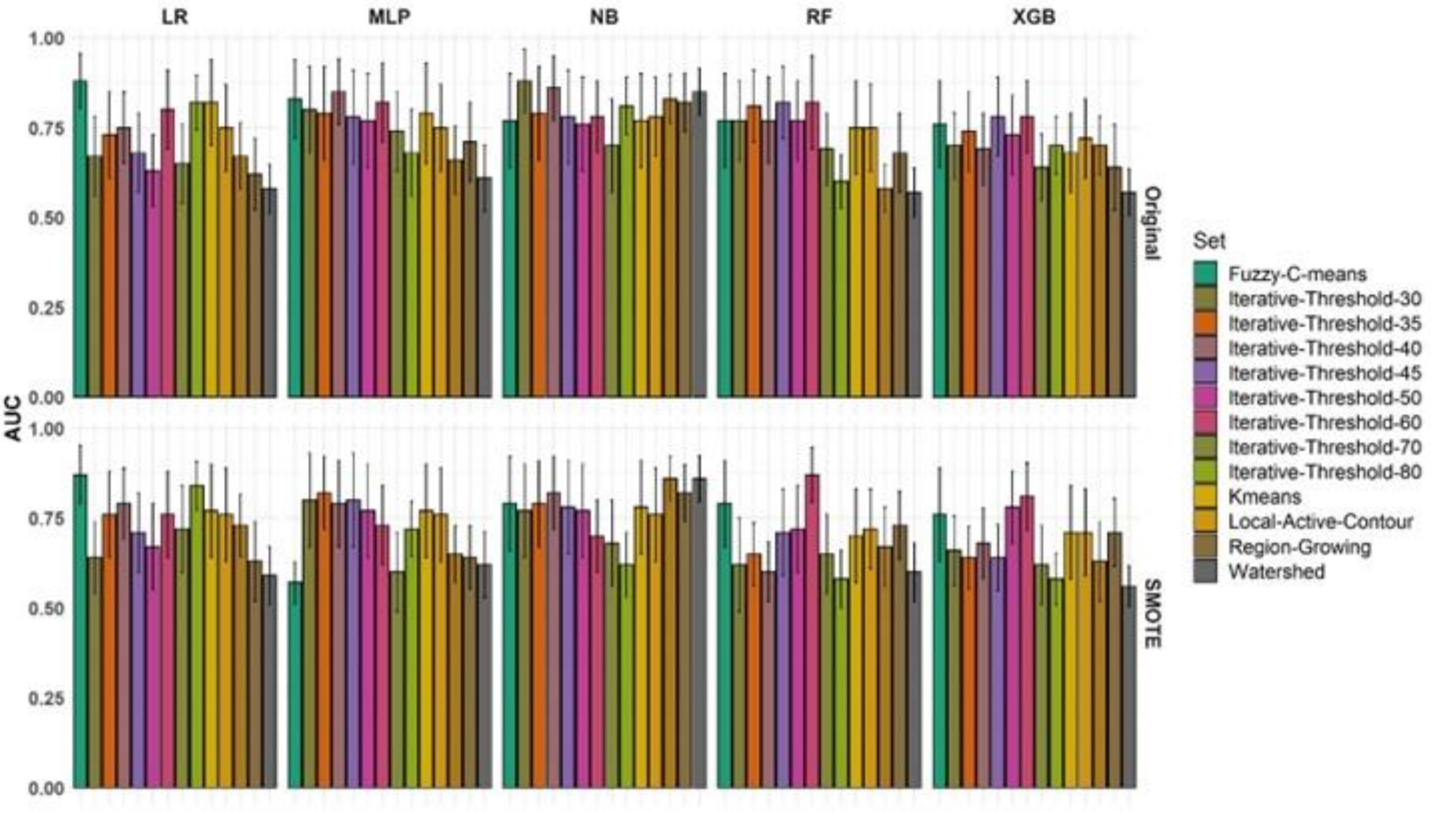
LR
MLP
NB
RF
XGB
AUC
1.00
0.75
0.50
0.25
0.00
Original
SMOTE
Set
Fuzzy-C-means
Iterative-Threshold-30
Iterative-Threshold-35
Iterative-Threshold-40
Iterative-Threshold-45
Iterative-Threshold-50
Iterative-Threshold-60
Iterative-Threshold-70
Iterative-Threshold-80
Kmeans
Local-Active-Contour
Region-Growing
Watershed

A

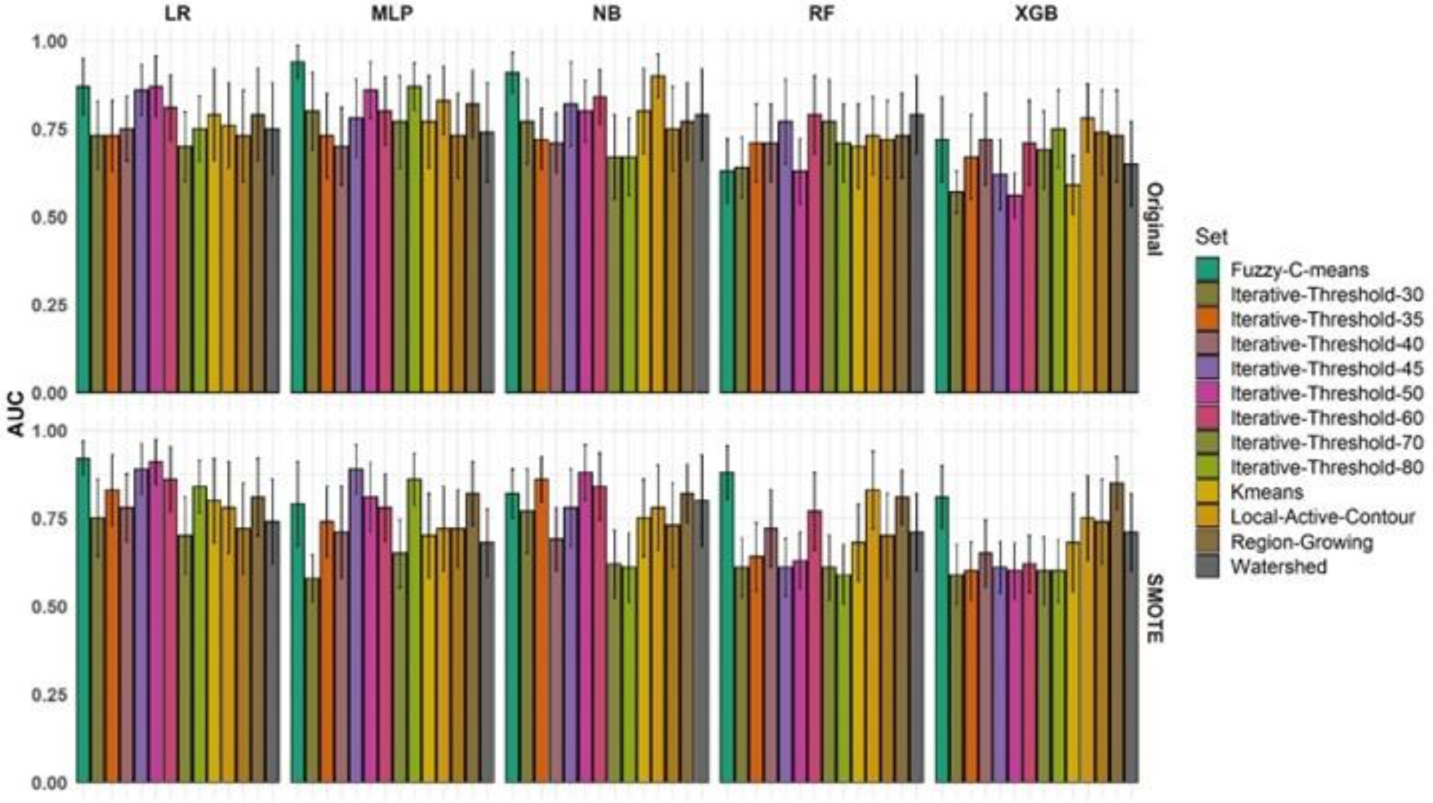
LR
MLP
NB
RF
XGB
AUC
1.00
0.75
0.50
0.25
0.00
Original
SMOTE
Set
Fuzzy-C-means
Iterative-Threshold-30
Iterative-Threshold-35
Iterative-Threshold-40
Iterative-Threshold-45
Iterative-Threshold-50
Iterative-Threshold-60
Iterative-Threshold-70
Iterative-Threshold-80
Kmeans
Local-Active-Contour
Region-Growing
Watershed

B

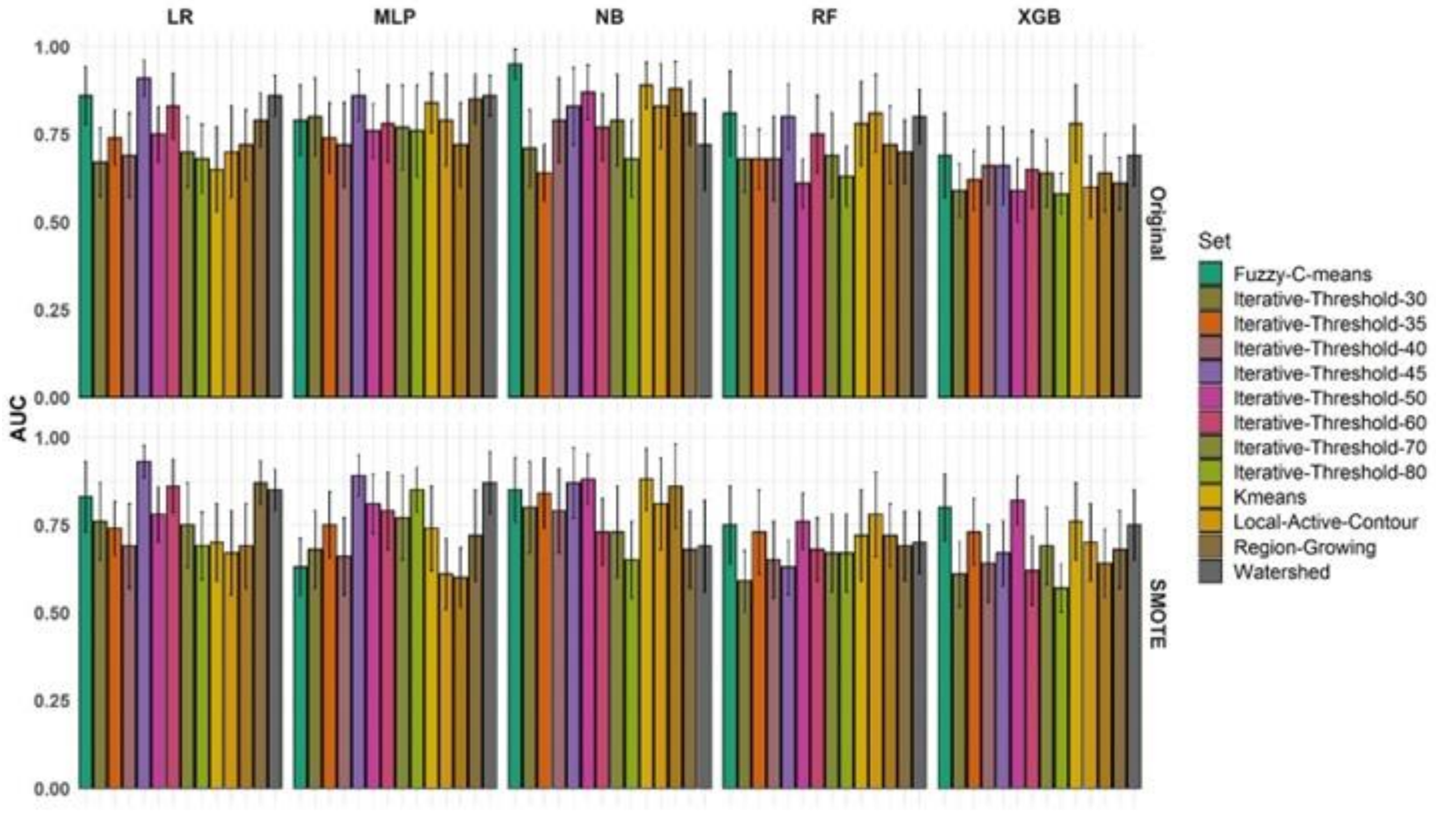

C

Figure 2. The calculated area under the curve (AUC) belonging to Boruta (A), MRMR (B), and RFE (C) feature selectors were applied to 13 image segmentation methods, including LAC, FCM, K means, Watershed, RG, and Iterative threshold besides 5 machine learning classifiers including MLP, LR, XGB, NB, and RF. Results are reported both with conducting SMOTE (lower plots) and original without SMOTE (upper plots).

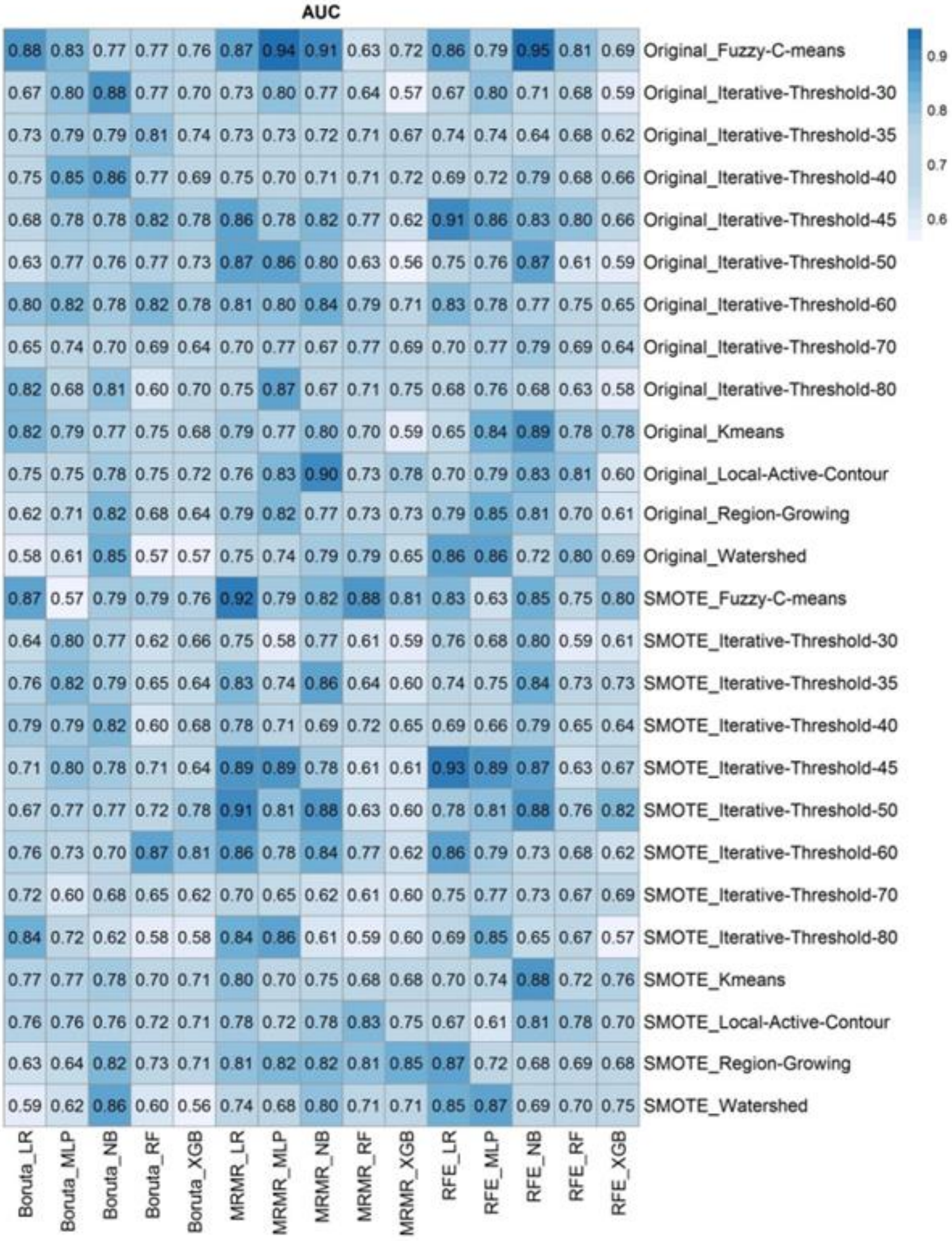

Figure 3. Predicting power (AUC) heatmap of multiple machine learning algorithms and feature selections over 13 image segmentations methods, including LAC, FCM, K means, Watershed, RG, and iterative thresholding besides 5 machine learning classifiers including MLP, LR, XGB, NB, and RF.

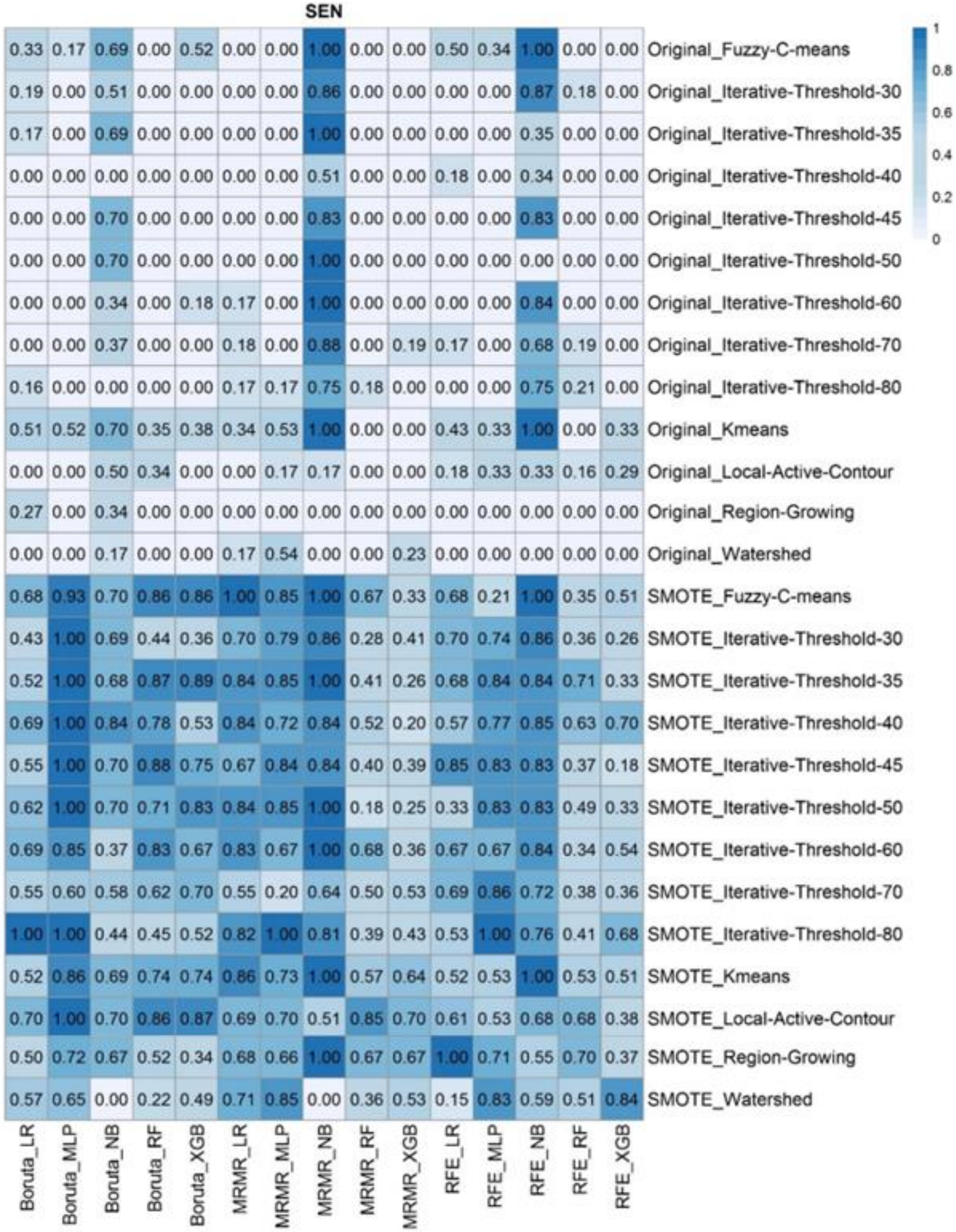

Figure 4. Sensitivity (SEN) heatmap of multiple machine learning algorithms and feature selections over 13 image segmentations methods, including LAC, FCM, K means, Watershed, RG, and iterative thresholding besides 5 machine learning classifiers including MLP, LR, XGB, NB, and RF.

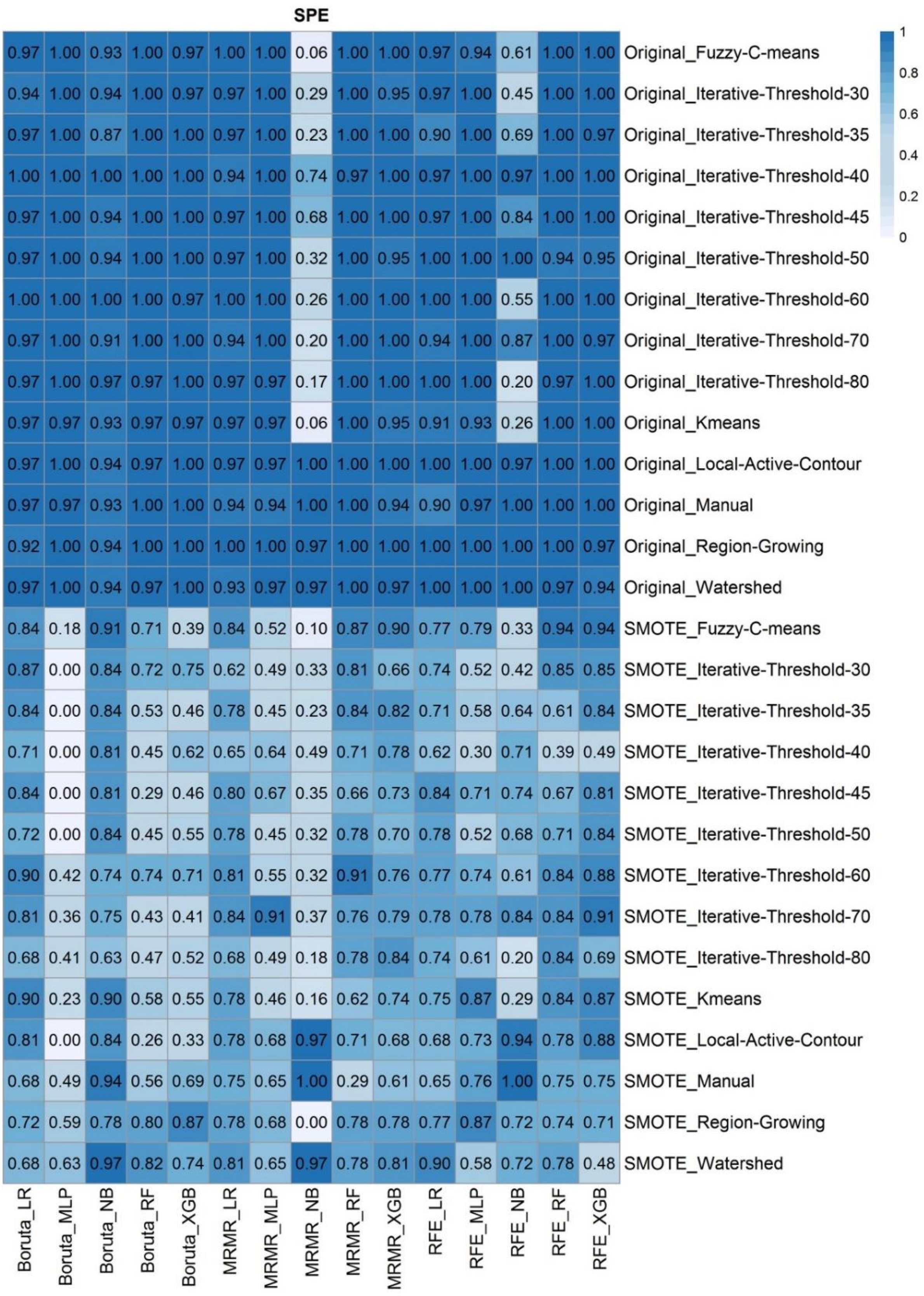

Figure 5. Specificity (SPE) heatmap of multiple machine learning algorithms and feature selections over 13 image segmentations methods, including LAC, FCM, K means, Watershed, RG, and iterative thresholding besides 5 machine learning classifiers including MLP, LR, XGB, NB, and RF.

Table 3. Selected features based on the combination of IT segmentation with a 45% threshold and RFE feature selection.

Category	Feature Name
Shape	Flatness
Shape	Maximum3D_Diameter
Shape	MajorAxis
First-order	Maximum
First-order	90Percentile
First-order	Range
First-order	RobustMeanAbsoluteDeviation
First-order	MeanAbsoluteDeviation
First-order	RootMeanSquared
First-order	10Percentile
First-order	Median
First-order	Mean
First-order	Minimum
First-order	Variance
First-order	InterquartileRange
Texture	GLSZM SmallAreaLowGrayLevelEmphasis
Texture	GLSZM SmallAreaHighGrayLevelEmphasis
Texture	GLDM LargeDependenceEmphasis
Texture	GLSZM SmallAreaEmphasis
